# Supplementary material for: Improved tropoelastin synthesis in the skin by codon optimization and nucleotide modification of tropoelastin-encoding synthetic mRNA
Source: Mol Ther Nucleic Acids. 2023 Aug 2;33:642–54. doi: 10.1016/j.omtn.2023.07.035 (PMC10462787; doi:10.1016/j.omtn.2023.07.035)
Supplement: Document S1. Figures S1–S7 and Table S2 [file mmc1.pdf]

OMTN, Volume *33*

Supplemental information

Improved tropoelastin synthesis in the skin by codon optimization and nucleotide modification of tropoelastin-encoding synthetic mRNA

Sonia Golombek, Thomas Hoffmann, Ludmilla Hann, Markus Mandler, Sabine Schmidhuber, Josefin Weber, Young-Tae Chang, Roman Mehling, Andrea Ladinig, Christian Knecht, Johanna Leyens, Christian Schlensak, Hans Peter Wendel, Achim Schneeberger, and Meltem Avci-Adali

SUPPLEMENTAL DATA

Table S1: Sequences of TE coding sequences

Table S2: Ranking list of in vitro tested TE mRNA variants.

Ranking	TE mRNA variant	Elastin [ng/mL]	Viability [%]
**1**	14_me^{1}Ψ/m5C	7381	71.66
**2**	14_me^{1}Ψ/C	6522	61.37
**3**	3_me^{1}Ψ/m5C	5540	70.08
**4**	native_me^{1}Ψ/C	4548	57.84
**5**	native_me^{1}Ψ/m5C	2566	62.86
**6**	4_me^{1}Ψ/C	2563	60.43
**7**	1_me^{1}Ψ/C	2127	69.97
**8**	3_me^{1}Ψ/C	2021	58.33
**9**	14_Ψ/m5C	1106	42.44
**10**	3_Ψ/m5C	887.4	40.75
**11**	native_Ψ/m5C	324.2	42.97
**12**	4_me^{1}Ψ/m5C	216.9	79.18
**13**	native_unmod	33.84	1.60
**14**	3_unmod	29.05	1.1
**15**	4_unmod	28.54	1.59
**16**	1_unmod	26.36	3.01
**17**	4_Ψ/m5C	22.09	50.28
**18**	14_unmod	20.75	1.41
**19**	1_me^{1}Ψ/m5C	15.25	77.24
**20**	1_Ψ/m5C	0	49.73

In vitro analysis of TE protein expression over 72 h

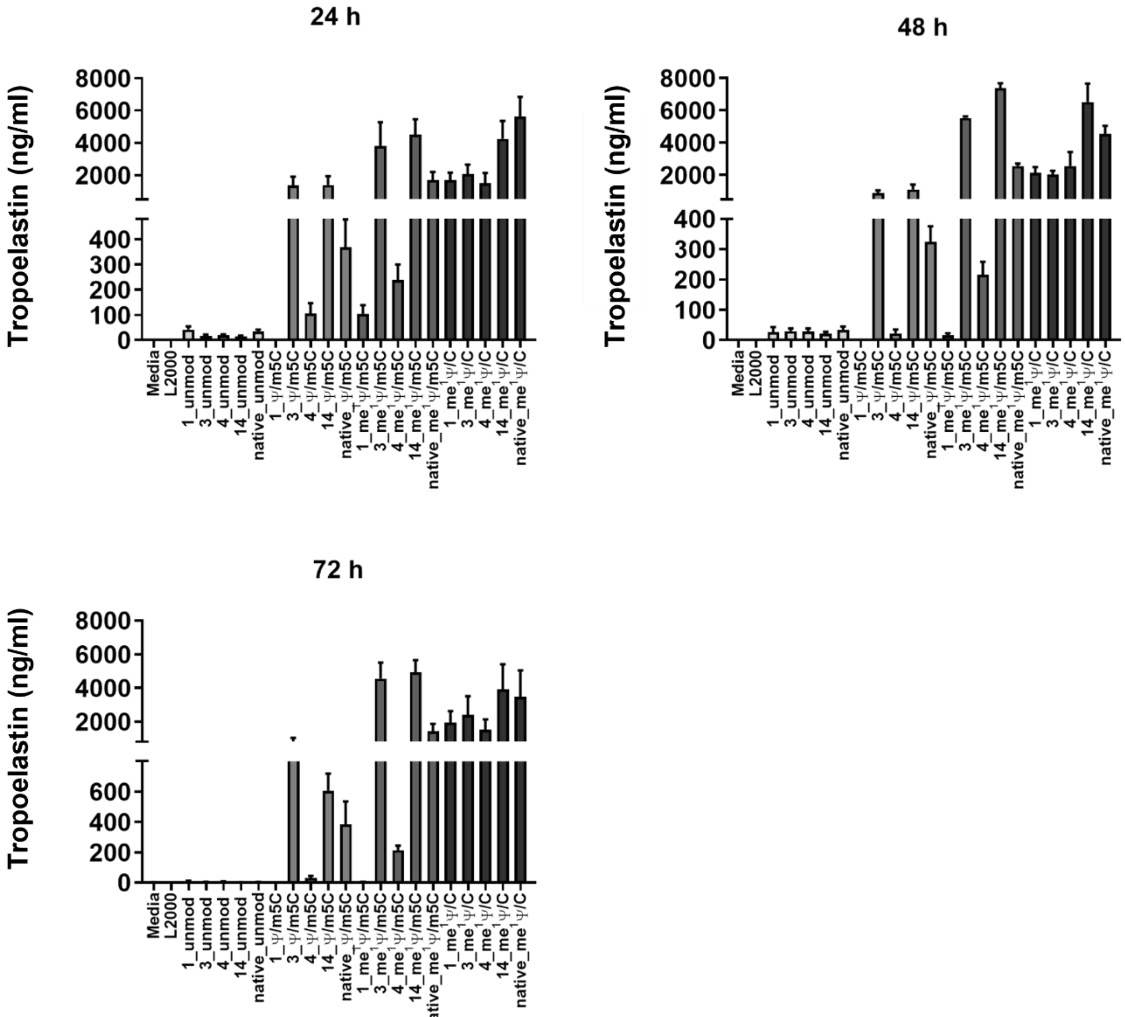

Figure S1: Detection of TE production over time*.* The influence of different TE mRNA variants on TE synthesis was tested by elastin ELISA. 3x10^5 EA.hy926 cells were transfected with 2.5 µg TE mRNA complexed with 4 µl Lipofectamine 2000 in OptiMEM at 37°C and 5% CO_2 for 4 h. Thereafter, the transfection complexes were replaced with cell culture medium, and the cells were incubated at 37°C and 5% CO_2 without further medium change. The elastin concentration was determined in cell supernatants after 24, 48, and 72 h. As controls, cells were treated with Lipofectamine 2000 (L2000) or OptiMEM only (Medium). The elastin concentration was determined in cell supernatants after 24, 48, and 72 h. Results are shown as mean + SEM (n = 3).

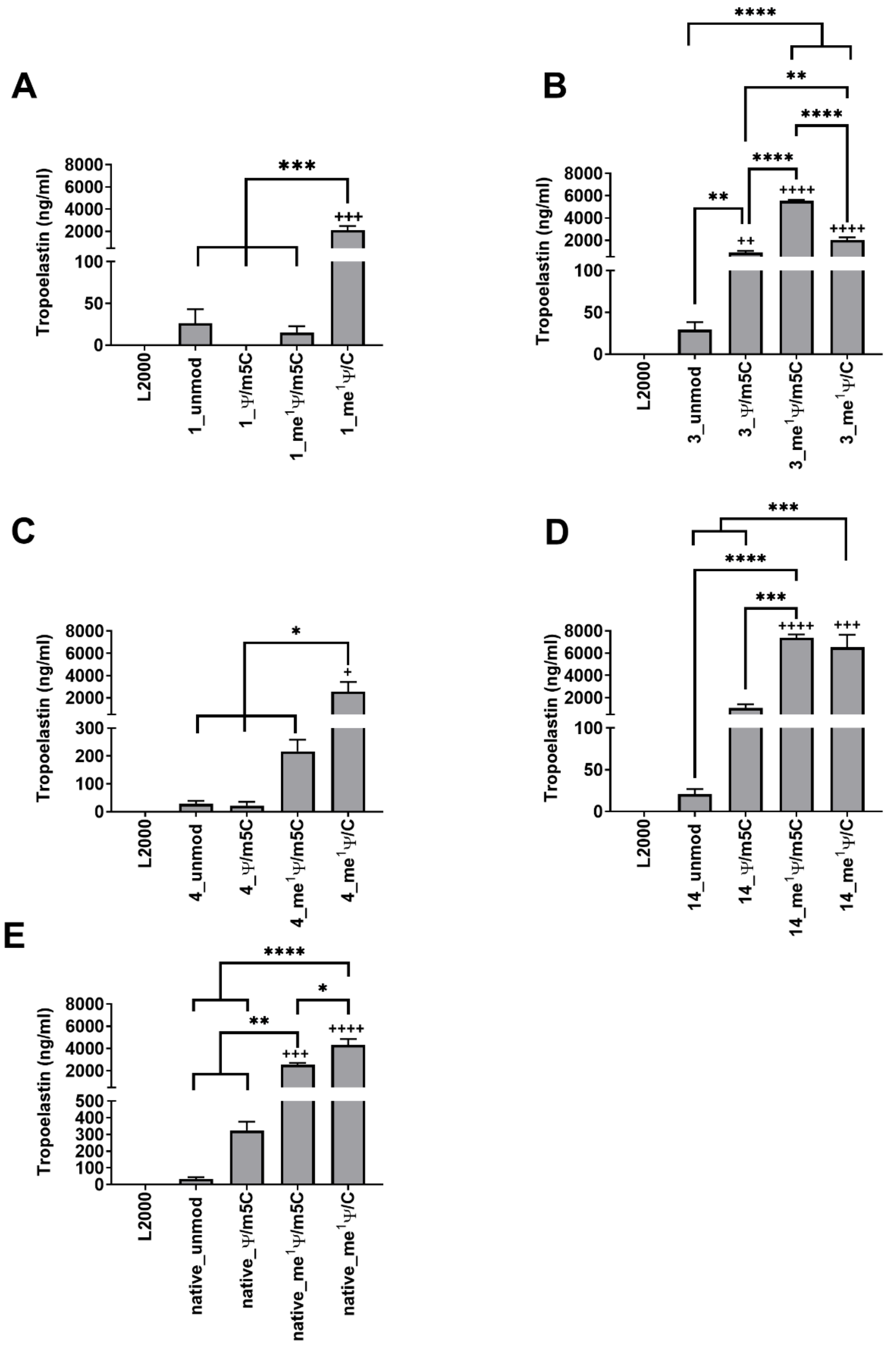

Figure S2: Analysis of TE synthesis after the delivery of TE mRNA into cells. 3x10^5 EA.hy926 cells were transfected with 2.5 µg TE mRNA complexed with 4 µl of Lipofectamine 2000 in OptiMEM for 4 h at 37°C and 5% CO_2. Thereafter, the transfection complexes were replaced by cell culture medium, and the cells were incubated for 48 h at 37°C and 5% CO_2. As a control, cells were treated with

Lipofectamine2000 (L2000) alone. TE concentration was detected in supernatants of cells using elastin ELISA to determine the influence of nucleotide modifications of TE mRNA variants (**A**) 1, (**B**) 3, (**C**) 4, (**D**) 14, and (**E**) native on TE synthesis. The results are shown as mean + SEM (n = 3). Statistical differences were determined using one-way ANOVA followed by Bonferroni’s multiple comparisons test. (*p < 0.05, **p < 0.01, *** p <0.001, ****p < 0.0001); + = statistical differences to L2000 control ($^{+}$p < 0.05, $^{++}$p < 0.01, $^{+++}$p < 0.001, $^{++++}$p < 0.0001).

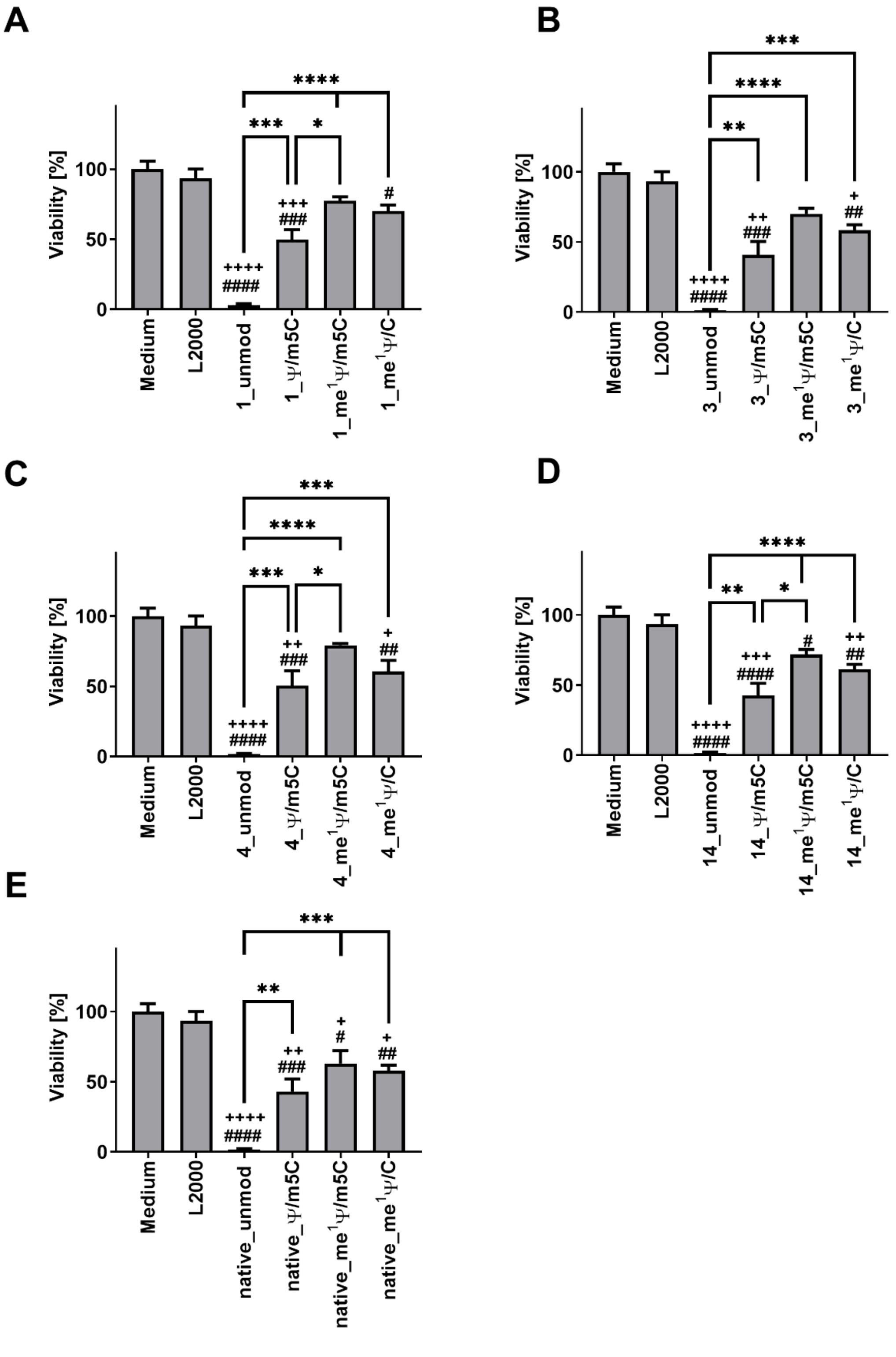

Figure S3: Influence of TE mRNAs on cell viability. $3x10^5$ EA.hy926 cells were transfected with 2.5 µg TE mRNA variants complexed with 4 µl of Lipofectamine 2000 (L2000) in OptiMEM for 4 h at 37°C and 5% CO_2. Thereafter, the transfection complexes were replaced by cell culture medium, and the cells were incubated at 37°C and 5% CO_2. After 24 h, the influence of nucleotide modifications of TE mRNA

variants (**A**) 1, (**B**) 3, (**C**) 4, (**D**) 14, and (**E**) native on cell viability was detected using Presto Blue assay. The viability of cells treated with OptiMEM (medium) was set to 100%. The results are shown as mean + SEM (n = 3). Statistical differences were determined using one-way ANOVA following Bonferroni's comparison test. (* p <0.05, ** p <0.01, *** p <0.001, ****p < 0.0001; # = statistical differences to the medium control ($^{\#}p < 0.05$, $^{\#\#}p < 0.01$, $^{\#\#\#}p < 0.001$, $^{\#\#\#\#}p < 0.0001$); + = statistical differences to L2000 control ($^{+}p < 0.05$, $^{++}p < 0.01$, $^{+++}p < 0.001$, $^{++++}p < 0.0001$).

Analysis of the presence of TE mRNA in the cells

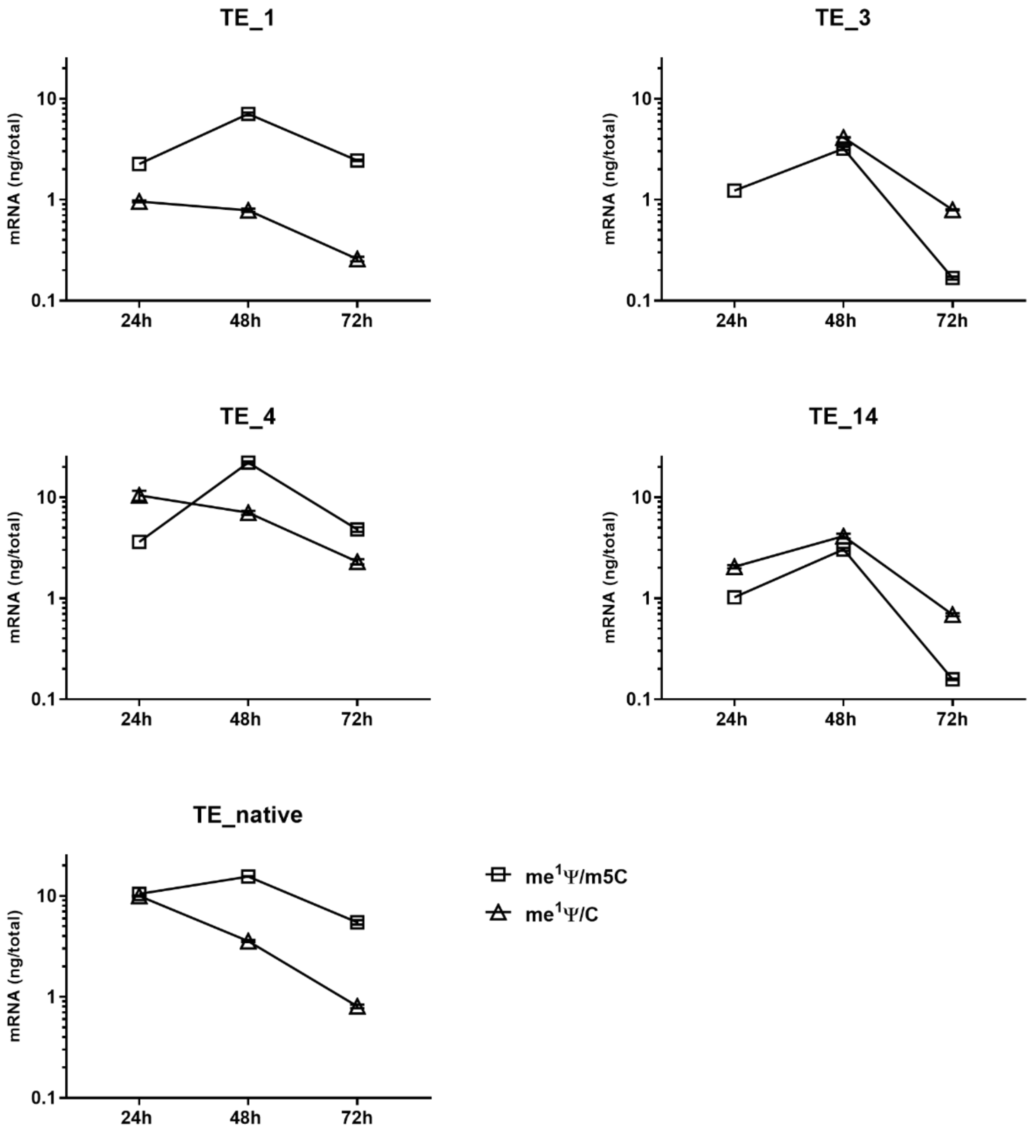

Figure S4: Analysis of the TE mRNA presence in EA.hy926 cells after transfection of TE mRNA variants. The effect of different TE mRNA sequence variants and nucleotide modifications on mRNA

decay was tested by qRT-PCR. Therefore, $3x10^5$ cells were transfected with 2.5 µg TE mRNA complexed with 4 µl of Lipofectamine 2000 in OptiMEM for 4 h at 37°C and 5% CO_2. Thereafter, the transfection complexes were replaced by cell culture medium, the cells were incubated for 2 h at 37°C, 5% CO_2, and the RNA was isolated. Total TE mRNA content was determined by qPCR. Results are shown as mean ± SEM (n = 3).

In vivo application of TE mRNA variants into pig skin by intradermal injection and macroscopic assessment of skin irritation

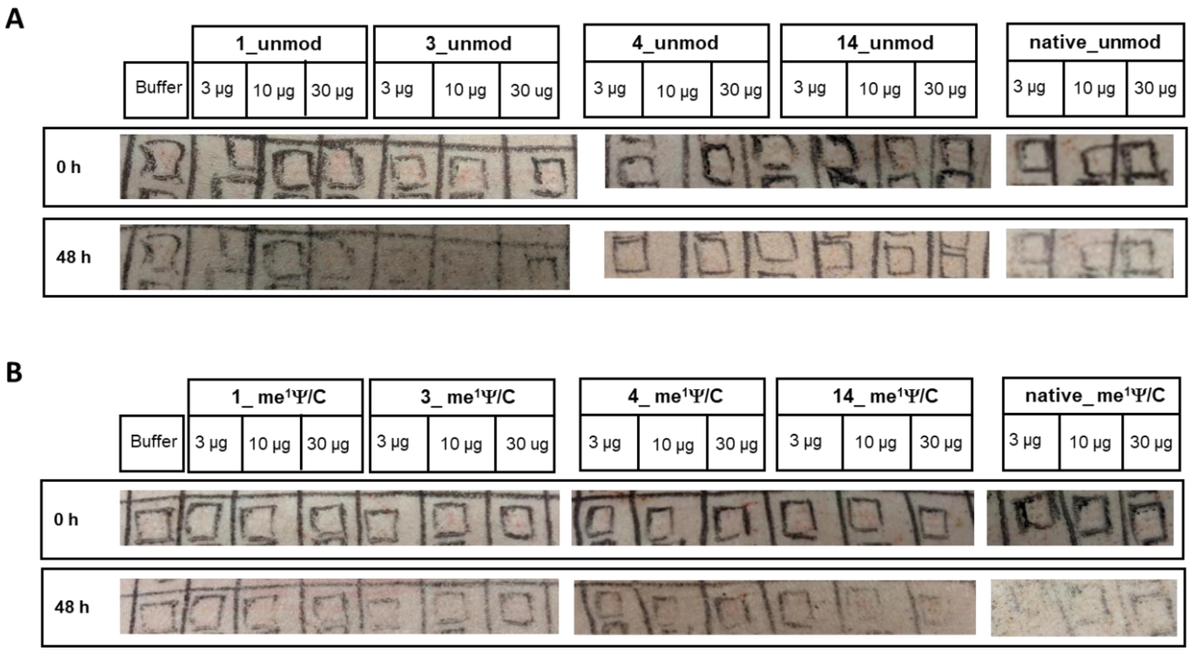

Figure S5: Representative photographic images of porcine skin after in vivo intradermal injection of TE mRNA variants. After injection of 9 x 10 µl Ringer´s lactate buffer without or with 3, 10, or 30 µg TE mRNA variants into a defined skin area of 1x1 cm, the injection sides were marked with a tattoo pen. **(A)** Injection sides of unmodified TE mRNA variants and **(B)** injection sides of $me^1\Psi$/C modified TE mRNA variants after 48 h are shown. No skin irritation or redness was visible.

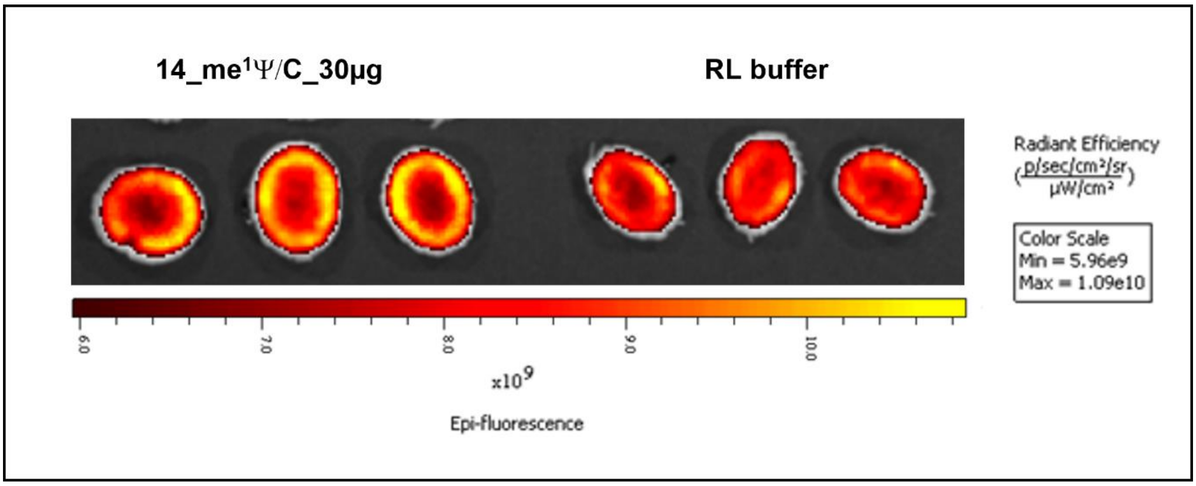

Figure S6: Representative image of IVIS detection of ElaNIR stained porcine skin samples 48 h after the intradermal injection of TE mRNA variants in vivo. 48 h post-injection, skin biopsies injected with 14_me[1]Ψ/C, corresponding RL buffer only were collected from the injection sites. Biopsies were stained with 20 µM ElaNIR for 30 min. Photographic images including a fluorescent heat map were acquired to indicate fluorescence intensity and distribution areas. Fluorescence emission in a defined region of interest (ROI) was normalized to photons per second per square centimeter per steradian and expressed as average radiant efficiency [p/s/cm2/sr]/[µW/cm^2].

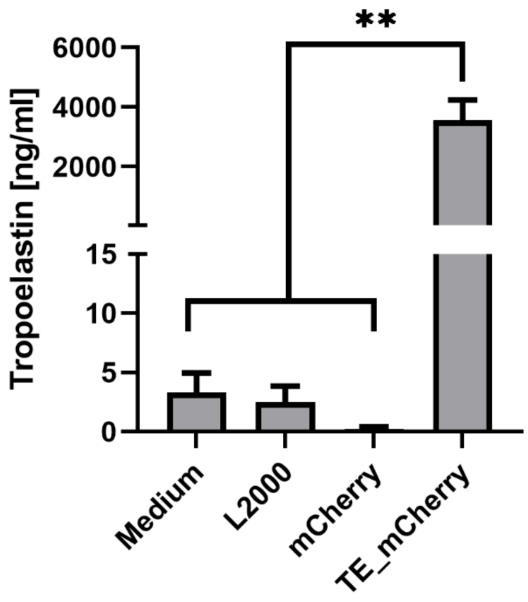

Figure S7: Detection of TE production after TE_mCherry transfection. 3x10^5 EA.hy926 cells were transfected with 2.5 µg mCherry or TE_mCherry mRNA complexed with 4 µl Lipofectamine 2000 (L2000) in OptiMEM at 37°C and 5% CO_2. After 4 h, transfection complexes were replaced with cell

culture medium and the cells were incubated at 37°C and 5% CO_2 for 24 h. Thereafter, the TE concentration was determined in cell culture supernatants using ELISA. As controls, cells were treated with Lipofectamine 2000 (L2000) or OptiMEM only (Medium). Results are shown as mean + SEM (n = 3). Statistical differences were determined using one-way ANOVA following Tukey's comparison test. (** $p < 0.01$).
