# Supplementary material for: Improved tropoelastin synthesis in the skin by codon optimization and nucleotide modification of tropoelastin-encoding synthetic mRNA
Source: Mol Ther Nucleic Acids. 2023 Aug 2;33:642–54. doi: 10.1016/j.omtn.2023.07.035 (PMC10462787; doi:10.1016/j.omtn.2023.07.035)
Supplement: Document S2. Article plus supplemental information [file mmc3.pdf]

Molecular Therapy
Nucleic Acids
Original Article

Improved tropoelastin synthesis in the skin by codon optimization and nucleotide modification of tropoelastin-encoding synthetic mRNA

Sonia Golombek,[1] Thomas Hoffmann,[2] Ludmilla Hann,[1] Markus Mandler,[2] Sabine Schmidhuber,[2] Josefin Weber,[1] Young-Tae Chang,[3] Roman Mehling,[4] Andrea Ladinig,[5] Christian Knecht,[5] Johanna Leyens,[1] Christian Schlensak,[1] Hans Peter Wendel,[1] Achim Schneeberger,[2] and Meltem Avci-Adali[1]

[1]Department of Thoracic and Cardiovascular Surgery, University Hospital Tübingen, Calwerstraße 7/1, 72076 Tübingen, Germany; [2]Accanis Biotech, Karl-Farkas-Gasse 22, Vienna 1030, Austria; [3]Department of Chemistry, Pohang University of Science and Technology (POSTECH), Pohang 37673, Republic of Korea; [4]Werner Siemens Imaging Center, Department of Preclinical Imaging and Radiopharmacy, Eberhard Karls University, Röntgenweg 13, 72076 Tübingen, Germany; [5]University Clinic for Swine, Department of Farm Animals and Veterinary Public Health, University of Veterinary Medicine, Veterinärplatz 1, Vienna 1210, Austria

Loss of elastin due to aging, disease, or injury can lead to impaired tissue function. In this study, *de novo* tropoelastin (TE) synthesis is investigated *in vitro* and *in vivo* using different TE-encoding synthetic mRNA variants after codon optimization and nucleotide modification. Codon optimization shows a strong effect on protein synthesis without affecting cell viability *in vitro*, whereas nucleotide modifications strongly modulate translation and reduce cell toxicity. Selected TE mRNA variants (3, 10, and 30 μg) are then analyzed *in vivo* in porcine skin after intradermal application. Administration of 30 μg of native TE mRNA with a me^1 Ψ modification or 10 and 30 μg of unmodified codon-optimized TE mRNA is required to increase TE protein expression *in vivo*. In contrast, just 3 μg of a codon-optimized TE mRNA variant with the me^1 Ψ modification is able to increase protein expression. Furthermore, skin toxicity is investigated *in vitro* by injecting 30 μg of mRNA of selected TE mRNA variants into a human full-thickness skin model, and no toxic effects are observed. Thereby, for the first time, an increased dermal TE synthesis by exogenous administration of synthetic mRNA is demonstrated *in vivo*. Codon optimization of a synthetic mRNA can significantly increase protein expression and therapeutic outcome.

INTRODUCTION

A milestone in the research of synthetic messenger RNA (mRNA)-based agents was achieved with the development and US Food and Drug Administration (FDA) approval of mRNA-based vaccines to combat the coronavirus pandemic. The potential of synthetic mRNA-based vaccines has been extensively studied in recent years for infectious disease prevention and cancer prophylaxis. However, the scope of application is not limited to immunotherapy. Synthetic mRNAs can also be used for protein replacement therapies to treat diseases and for tissue regeneration.[1]

In contrast to plasmid DNA or viral vectors, synthetic mRNAs offer several advantages: they can be produced by rapid and simple *in vitro* transcription (IVT). The synthetic mRNA does not need to enter the nucleus, and consequently, no integration into the host genome occurs, which greatly reduces the risk of mutations. Due to natural physiological decay, synthetic mRNAs are transiently present in cells, avoiding the side effects associated with protein overexpression. Furthermore, synthetic mRNAs can be more easily delivered into cells due to their smaller size compared with plasmids and viral vectors.[2–4]

Elastin is one of the most important proteins of the extracellular matrix (ECM) and provides elasticity and flexibility to organs such as the lungs, heart, blood vessels, and skin.[5,6] The half-life of elastin is approximately 74 years,[7] and it is one of the most stable proteins known. Elastin is produced and secreted as a soluble monomer tropoelastin (TE) by various cell types, such as smooth muscle cells, fibroblasts, and endothelial cells.[8] However, TE expression is largely restricted to the third trimester of fetal development and the early postnatal years. From adolescence onward, elastin synthesis decreases and ceases in adults.[9] Thus, the persistence of elastin produced during development is important for the proper functioning of elastic connective tissues.

Although elastin is a highly resilient component of the ECM, elastolysis may occur with age and under certain pathophysiological conditions. The degeneration of elastic fiber can lead to dysfunction and the release of elastin-derived peptides (EDPs).[10] Some of these EDPs, known as elastokines, have been shown to have bioactive properties

Received 14 November 2022; accepted 31 July 2023;
https://doi.org/10.1016/j.omtn.2023.07.035.

Correspondence: Meltem Avci-Adali, Department of Thoracic and Cardiovascular Surgery, University Hospital Tübingen, Calwerstraße 7/1, 72076 Tübingen, Germany.
E-mail: meltem.avci-adali@uni-tuebingen.de

 © 2023 The Authors.
This is an open access article under the CC BY license (http://creativecommons.org/licenses/by/4.0/).

Table 1. Codon-optimized-sequence TE mRNA variants

Sequence variant	CAI	GC content
TE_1	0.9080	76.4%
TE_3	0.8010	72.2%
TE_4	0.7532	66.3%
TE_14	0.7200	62.2%
TE_native	0.6947	64.3%

involved in the development of cardiovascular diseases.[11] Further studies suggest that they may play a role in the pathogenesis of Alzheimer's disease by leading to amyloid deposition.[12] However, the mechanism by which EDPs act in the nervous system is still mostly unclear, and further research is needed to fully understand their impact.[13]

Elastic fibers are composed of an insoluble inner core of elastin, which accounts for ~90% of mature fibers, and fibrillin-rich microfibrils.[14] The formation of elastic fibers, so-called elastogenesis, is a highly complex process[15] composed of the secretion of TE, followed by coacervation, deposition onto microfibrils providing a structural scaffold for the deposition of TE, and the covalent cross-linking of lysine residues by lysyl oxidase.[16]

Genetic diseases, such as Williams-Beuren syndrome (WBS) or cutis laxa, result in impaired elastinogenesis and lead to loose skin and vascular defects, such as supravalvular aortic stenosis.[17] In the skin, the damage of elastin fibers caused by injuries, diseases, sunburn, and age-related degradation results in irreversible loss of skin elasticity due to the lack of repair mechanisms and TE synthesis. The loss of elastin in the dermis after severe burns results in significant physical damage such as scarring, wound contraction, and loss of skin extensibility.[18] The regeneration of elastic fibers has been shown to play a crucial role in the process of wound healing and scar formation, as well as in restoring the functionality and elasticity of the skin.[18,19] In our recently published study, we successfully demonstrated an increased elastin synthesis in various human cell types, including mesenchymal stem cells isolated from a WBS patient with elastin deficiency, after transfection with synthetic TE-encoding mRNA, demonstrating the applicability of synthetic TE-encoding mRNA for *de novo* synthesis of elastin.[20] In addition, a significantly increased amount of elastin was detected in *ex vivo* porcine skin after intradermal microinjection of synthetic TE mRNA.

In this study, we aimed to investigate whether the production of TE can be further improved by codon optimization and nucleotide modification of the native TE mRNA. The impact of these modifications on cell viability was analyzed *in vitro* to identify the TE mRNA candidate with the highest protein expression efficiency and the lowest cell toxicity. The selected TE mRNA variants were then analyzed in a large animal model after injection into the skin of pigs. In addition, the skin toxicity was evaluated by injection into a human skin model.

RESULTS

In silico optimization of the TE mRNA sequence

The coding sequence (CDS) of TE was selected considering the GC content and the human codon adaptivity index (CAI), which measures the percentage of codons that are the most abundant choice in any organism, with CAI values ranging from 0 to 1. From a large number of TE sequences, four codon-optimized TE mRNA variants (Table S1) were selected, and their translation into TE protein was tested compared with native human TE mRNA (Table 1). TE is an especially difficult protein to optimize, because 76% of all of its amino acids comprise only glycine (29%), alanine (22%) valine (13%), and proline (12%). All of these four amino acids have very GC-rich codons, and therefore, the overall GC content of the mRNA is very high.

To analyze the influence of different codon-optimized TE mRNA variants (Table 1) and varying nucleotide modifications on translation efficiency, five different TE mRNA variants with unmodified nucleotides cytidine and uridine (CTP/UTP) or with modifications of pseudouridine (Ψ) and 5-methylcytidine (m5C) (Ψ/m5C), N1-methylpseudouridine (me^1 Ψ) and 5-methylcytidine (me^1 Ψ/m5C), or N1-methylpseudouridine and cytidine (me^1 Ψ/C) (Table 2) were produced.

Codon optimization of TE mRNA highly affected the translation efficiency without influencing the cell viability *in vitro*

To analyze the influence of TE codon sequence variations on translation into TE as well as on cell viability, 3×10^5 EA.hy926 cells were transfected with 2.5 μg TE mRNA complexed with 4 μL Lipofectamine 2000 (L2000) in OptiMEM. After 24, 48, and 72 h, supernatants were collected, and TE concentration was detected using ELISA. Increased TE production was already detected after 24 h; however, quantitatively, the highest TE amounts were measured 48 h post-transfection (Figure S1). Cells treated with L2000 alone served as controls. The influence of codon optimization (Figure 1) of the TE mRNA or nucleotide modification (Figure S2) of TE mRNA variants on TE protein expression was analyzed.

The TE mRNA variant 14 resulted in the highest TE protein expression (Figure 1) for all nucleotide modifications. After the transfection of cells with unmodified TE mRNA (unmod), only very low amounts of TE were detected in the supernatants (Figure 1A), which could be caused by the high cytotoxic effect of the unmodified mRNA *in vitro* (Figure S3A). The modification of TE mRNA variant 3 with Ψ/m5C (Figure 1B) or me^1 Ψ/m5C (Figure 1C) resulted in significantly higher protein expression compared with the control (L2000). The highest protein expression was detected when mRNA variant 3 was modified with me^1 Ψ/m5C. The TE mRNA variant 14 led to significantly increased TE protein expression when it was modified with Ψ/m5C (Figure 1B), me^1 Ψ/m5C (Figure 1C), or me^1 Ψ/C (Figure 1D), with the highest protein expression when modified with me^1 Ψ/m5C (Figure 1C). Both me^1 Ψ/m5C (Figure 1C) and me^1 Ψ/C (Figure 1D) modifications of the native mRNA resulted in significantly higher TE protein expression compared with the control, and the me^1 Ψ/C modification yielded the highest protein expression.

Table 2. Nucleotide modifications of TE mRNA variants

	Nucleotide modifications			
Sequence variant	CTP/UTP	Ψ/m5C	me[1] Ψ/m5C	me[1] Ψ/C
1	1_unmod	1_Ψ/m5C	1_me[1]Ψ/m5C	1_me[1]Ψ/C
3	3_unmod	3_Ψ/m5C	3_me[1]Ψ/m5C	3_me[1]Ψ/C
4	4_unmod	4_Ψ/m5C	4_me[1]Ψ/m5C	4_me[1]Ψ/C
14	14_unmod	14_Ψ/m5C	14_me[1]Ψ/m5C	14_me[1]Ψ/C
Native	native_unmod	native_Ψ/m5C	native_me[1] Ψ/m5C	native_me[1] Ψ/C
TE_mCherry	–	–	–	native_mCherry_me[1] Ψ/C

Five different sequence variants were used for the synthesis of TE-encoding mRNA with different nucleotide modifications and TE_mCherry mRNA. unmod, unmodified; Ψ, pseudo-UTP; m5C, 5-methyl-CTP; me[1] Ψ, N[1]-methylpseudo-UTP.

The influence of codon optimization (Figure 2) of TE mRNA or nucleotide modification (Figure S3) of TE mRNA variants on cell viability was analyzed 24 h post-transfection using the PrestoBlue assay. Cells treated with either medium or L2000 served as controls. Regardless of sequence variant, the unmodified mRNAs exhibited high cytotoxicity to cells compared with their nucleotide-modified variants (Figure 2A). Surprisingly, the nucleic acid sequence variations did not affect cell viability when the same nucleotide modification was used. The highest cell viability of up to 79% was observed after the transfection of cells with TE mRNA variants modified with me[1] Ψ/m5C (Figure 2C), followed by me[1] Ψ/C (Figure 2D) and Ψ/m5C (Figure 2B) modified TE mRNA variants.

Nucleotide modifications of codon-optimized TE mRNA variants strongly modulated translation efficiency *in vitro* and reduced cellular toxicity

The nucleotide modifications of TE mRNA variants had a strong effect on the translation of the mRNA variants as shown by the produced TE protein amounts in Figure S2. In particular, the incorporation of me[1] Ψ/m5C or me[1] Ψ/C nucleotides had a beneficial impact on TE protein expression. In the case of TE mRNA variants 1 (Figure S2A) and 4 (Figure S2C), only the incorporation of me[1] Ψ/C into the mRNA resulted in significantly increased TE protein expression compared with the control (L2000). Significantly increased TE protein expression was also observed using the TE mRNA variant 3 with Ψ/m5C, me[1] Ψ/m5C, and me[1] Ψ/C modifications (Figure S2B). The TE mRNA variants 14 (Figure S2D) and native (Figure S2E) generated using me[1] Ψ/m5C or me[1] Ψ/C significantly increased the amount of expressed TE protein compared with the control. The highest translation for TE variants 1, 4, and native was obtained by me[1] Ψ/C modification. In contrast, for TE variants 3 and 14, the highest TE protein expression was detected when using me[1] Ψ/m5C. These data indicate that, in addition to codon optimization, the nucleotide modification of each TE mRNA variant also has a strong influence on translation.

The influence of nucleotide modifications of the TE mRNA variants on cell viability was also analyzed (Figure S3). For all TE mRNA sequence variants (Figures 2A–2D), the use of me[1] Ψ/m5C and me[1] Ψ resulted in the highest cell viability compared with TE mRNA variants with unmodified nucleotides or Ψ/m5C modifications. Overall, cells transfected with TE mRNA containing the nucleotide modification me[1] Ψ/m5C showed the highest cell viability. Thus, the incorporation of me[1] Ψ/m5C or me[1] Ψ resulted in improved cell viability up to 30%.

The presence of transfected TE mRNA with these nucleotide modifications could be detected in cells up to 72 h after transfection (Figure S4). No differences in mRNA decay were detected between the different mRNA variants and nucleotide modifications.

In summary, the highest TE protein expression was detected with TE mRNA variant 14. Nucleotide modification of each TE mRNA variant resulted in increased protein expression, with the highest protein expression after the transfection with 14_me[1] Ψ/m5C or 14_me[1] Ψ/C. Surprisingly, codon optimization did not affect cell viability, but nucleotide modification did. The highest cell viability was observed with me[1] Ψ/m5C modification. In Table S2, the ranking of all 20 mRNA variants tested *in vitro* is shown. It should be considered that high protein expression is desirable, while toxicity should be as low as possible.

The *in vivo* administration of TE mRNA into porcine skin significantly increased TE protein expression

TE mRNA sequence variants with the me[1] Ψ nucleotide modifications showed reduced cell toxicity and increased protein expression efficiency along with stable mRNA presence as determined by mRNA decay analyses in cells. Therefore, unmodified or me[1] Ψ/C-modified TE mRNA variants native, 1, 3, 4, and 14, as well as me[1] Ψ/C-modified native TE_mCherry, were selected for screening in porcine skin to analyze protein expression efficiency *in vivo*.

No skin irritation was observed at the injection sites 48 h after injection (Figure S5). *De novo* synthesis of TE in porcine skin was determined 48 h after intradermal injection of TE mRNAs in Ringer's lactate (RL) buffer. Elastin-specific ElaNIR staining, which is well suited to detecting elastin in tissues, was used to determine elastin content in whole-skin biopsies. The ElaNIR-specific fluorescent

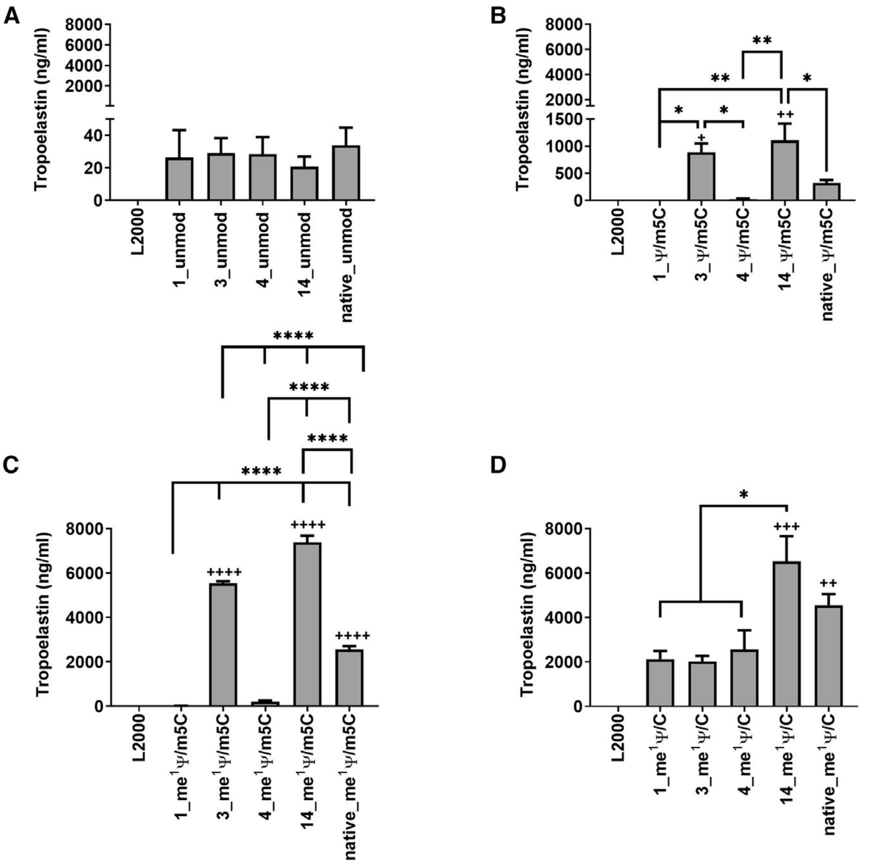

Figure 1. Analysis of TE synthesis after the delivery of TE mRNA into cells

EA.hy926 cells (3×10^5) were transfected with 2.5 μg TE mRNA complexed with 4 μL of Lipofectamine 2000 in OptiMEM for 4 h at 37°C and 5% CO_2. Thereafter, the transfection complexes were replaced by cell culture medium, and the cells were incubated for 48 h at 37°C and 5% CO_2. As a control, cells were treated with Lipofectamine 2000 (L2000) alone. The TE concentration was detected in supernatants of the cells using elastin ELISA to determine the influence of the different TE mRNA variants 1, 3, 4, 14, and native with (A) unmodified and (B) Ψ/m5C, (C) me^{1} Ψ/m5C, and (D) me^{1} Ψ/C modified nucleotides on TE synthesis was analyzed. The results are shown as the mean + SEM (n = 3). Statistical differences were determined using one-way ANOVA followed by Bonferroni's multiple comparisons test ($^{*}p < 0.05$, $^{**}p < 0.01$, $^{****}p < 0.0001$); statistical differences compared with the L2000 control are indicated by $^{++}p < 0.01$, $^{+++}p < 0.001$, $^{++++}p < 0.0001$.

signal was then measured using an *in vivo* imaging system (IVIS) (Figures 3A–3D).

The injections of 10 and 30 μg of unmodified TE mRNA variant 14 (Figure 3A) resulted in significantly higher fluorescent signal compared with the injection of RL buffer only. In the case of me^{1} Ψ/C-modified TE mRNA variant 14, application of just 3 μg of 14_me^{1} Ψ/C resulted in a significant increase in elastin levels, and increased elastin content was also observed after intradermal injection of 10 and 30 μg of 14_me^{1} Ψ/C (Figure 3B). A representative image of IVIS detection of ElaNIR-stained porcine skin samples 48 h after intradermal injection of TE mRNA variants *in vivo* is shown in Figure S6. Moreover, the injection of 30 μg of native_me^{1} Ψ/C resulted in significantly increased amounts of elastin in the skin (Figure 3C).

To more easily distinguish between endogenous and *de novo* synthesized elastin expressed after TE mRNA administration, a TE mRNA construct with an N-terminal mCherry-encoding tag sequence was designed and administered *in vivo*. The production of TE protein was confirmed after the transfection of EA.hy926 cells with 2.5 μg TE-mCherry mRNA (Figure S7). The expressed mCherry-tagged TE protein could be detected in skin biopsies after injection of 30 μg TE_mCherry mRNA and showed significantly increased fluorescence intensity compared with untreated skin controls (Figure 3D). Fluorescence microscopy images of the sectioned biopsies showed that the mCherry-tagged TE was distributed mainly in the dermis near the resident cells (nuclei stained with DAPI) (Figure 3E), but some fluorescent signal was also detected in the cornified layer, suggesting that some of the TE-mCherry mRNA could be taken up during the cornification process by the cells and remain intracellular after the translation.

No skin toxicity or intracellular innate immune activation was observed after the administration of TE mRNA variants in an *in vitro* human skin model

Potential toxic and immunogenic effects of TE mRNA variants in the skin were analyzed after intradermal application into human Phenion Full-Thickness (FT) skin models containing keratinocytes and fibroblasts. The TE mRNA variants with the highest protein expression efficiency *in vivo*, 14_me^{1} Ψ/C, 14_unmod, and native_me^{1} Ψ/C, and, in addition, native_Ψ/m5C were injected into the skin models (Figure 4A). Similar to the *in vivo* experiments, 30 μg TE mRNA in 90 μL RL buffer was injected. Only-RL-injected and untreated skin models served as controls. Cell viability in the skin models was determined 24 h post-injection using the 3-(4,5-dimethylthiazol-2-yl)-2,5-diphenyltetrazolium bromide (MTT) assay. Injection of the different TE mRNA variants showed no negative effects on cell viability (Figure 4B). In addition, 24 h post-injection, immune activation of the cells of the Phenion FT skin model was analyzed by quantitative reverse transcription-polymerase chain reaction (qRT-PCR). No significant increase in the expression of the immune activation markers IL-6, IL-8, CXCL-10, and interferon-β (IFN-β) was detected in the TE mRNA-treated groups

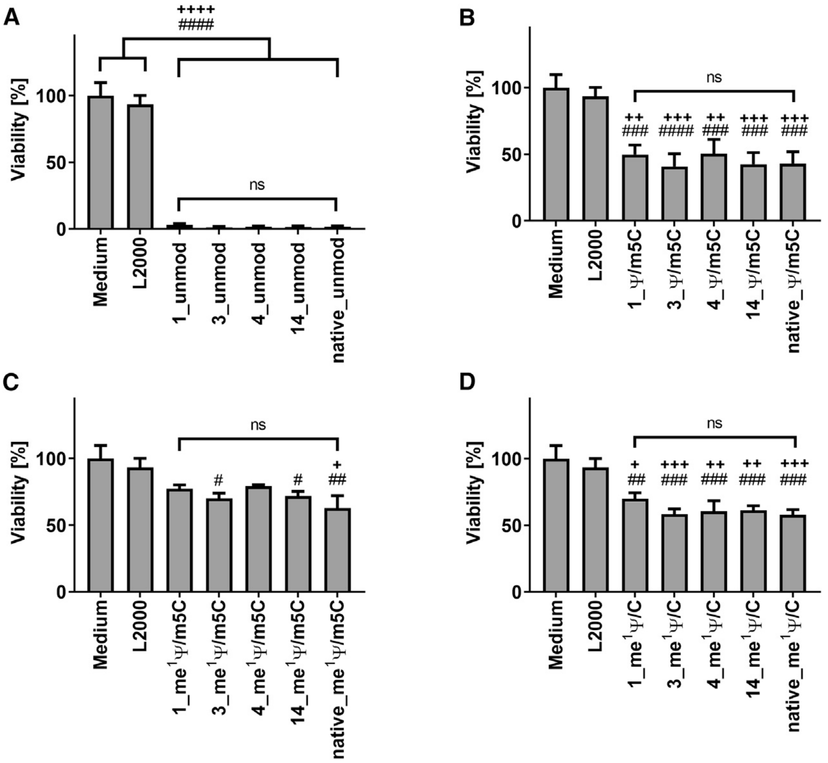

Figure 2. Influence of TE mRNAs on cell viability

EA.hy926 cells (3×10^5) were transfected with 2.5 μg TE mRNA variants complexed with 4 μL of Lipofectamine 2000 in OptiMEM for 4 h at 37°C and 5% CO_2. Thereafter, the transfection complexes were replaced by cell culture medium, and the cells were incubated at 37°C and 5% CO_2. The influence of the different TE mRNA sequence variants 1, 3, 4, 14, and native with (A) unmodified and (B) Ψ/m5C, (C) me^{1}Ψ/m5C, and (D) me^1 Ψ/C modified nucleotides on cell viability was analyzed 24 h after the transfection of cells using the PrestoBlue assay. The viability of cells treated with OptiMEM (medium) was set to 100%. The results are shown as the mean + SEM (n = 3). Statistical differences were determined using one-way ANOVA following Bonferroni's comparison test (ns, not significant); statistical differences compared with the medium control are indicated by $^{\#}p < 0.05$, $^{\#\#}p < 0.01$, $^{\#\#\#}p < 0.001$, $^{\#\#\#\#}p < 0.0001$, and statistical differences compared with the L2000 control are indicated by $^{+}p < 0.05$, $^{++}p < 0.01$, $^{+++}p < 0.001$, $^{++++}p < 0.0001$.

compared with the control or between the different TE mRNA groups (Figure 4C).

DISCUSSION

Elastin is responsible for the elasticity and resilience of the skin and allows the skin to stretch and return to its original shape.[21] It also plays an active role in regulating wound healing, contraction, and scar formation.[18] However, injury, genetic disorders, and aging can lead to elastin damage and irreversible loss of skin integrity and elasticity due to the lack of elastin turnover and physiological repair mechanisms. After an injury, the *de novo* elastin production in the skin may improve wound healing outcomes,[22] prevent scarring, and also restore skin elasticity in scars that have already formed. Therefore, new treatment strategies targeting the restoration of depleted elastin are of great clinical importance for the regeneration of damaged skin.[23]

In our previous study, we applied TE-encoding mRNA to induce elastin synthesis and demonstrated increased elastin synthesis *in vitro* and *ex vivo* in porcine skin.[20] In the present study, we highlight the importance of optimizing synthetic mRNA to achieve the best protein expression profiles. The *in vitro* screening of 20 different TE mRNAs was first performed with four different CDSs and five different nucleotide modifications with Ψ, m5C, me^1 Ψ, and unmodified nucleotides.

Not only the use of modified nucleotides could highly increase the mRNA translation efficiency, but also the usage of optimized codons. Subsequently, *de novo* synthesis of TE protein in porcine skin was demonstrated 48 h after the administration of synthetic TE mRNAs for the first time *in vivo*. Thereby, a synthetic TE mRNA variant (14_me^1 Ψ/C) was designed, which significantly improved TE protein expression in the skin after intradermal injection, without showing any skin irritation. Furthermore, for the first time, the *in vivo* TE synthesis following intradermal administration of synthetic TE mRNA was demonstrated in porcine skin.

In recent decades, various strategies have been applied to restore skin elasticity by inducing elastogenesis, e.g., the use of TGF-β[24,25] or insulin-like growth factor-1 (IGF-1).[26,27] Rothuizen and colleagues analyzed the effects of TGF-β, minoxidil, and IGF-1 in vascular cells at the transcriptional and translational levels. IGF-1 and minoxidil had little effect on TE mRNA expression, whereas TGF-β increased TE mRNA levels, but this increase did not affect protein levels.[27] Furthermore, it should be also noted that TGF-β can significantly promote tumor progression, invasion, and metastasis.[28]

Mithieux and Weiss tested the use of recombinant human TE (rhTE) protein for wound healing to increase the numbers and thickness of elastic fibers in full-thickness dermal substitutes containing the patient's dermal fibroblasts.[29] Repeated treatment of fibroblasts from 0-, 10-, 31-, and 51-year-old donors with rhTE protein resulted in the incorporation of TE into a growing elastic network. Without exogenous TE supplementation, there was no evidence of elastic fiber synthesis. In further studies, injection of rhTE cross-linked with derivatized hyaluronic acid (dHA) resulted in the production of colocalized human-rat elastic fibers *in vivo* in rats.[30] In another study, the use of an adenoviral vector carrying a TE gene resulted in successful transfection of vascular smooth muscle cells *in vitro* and

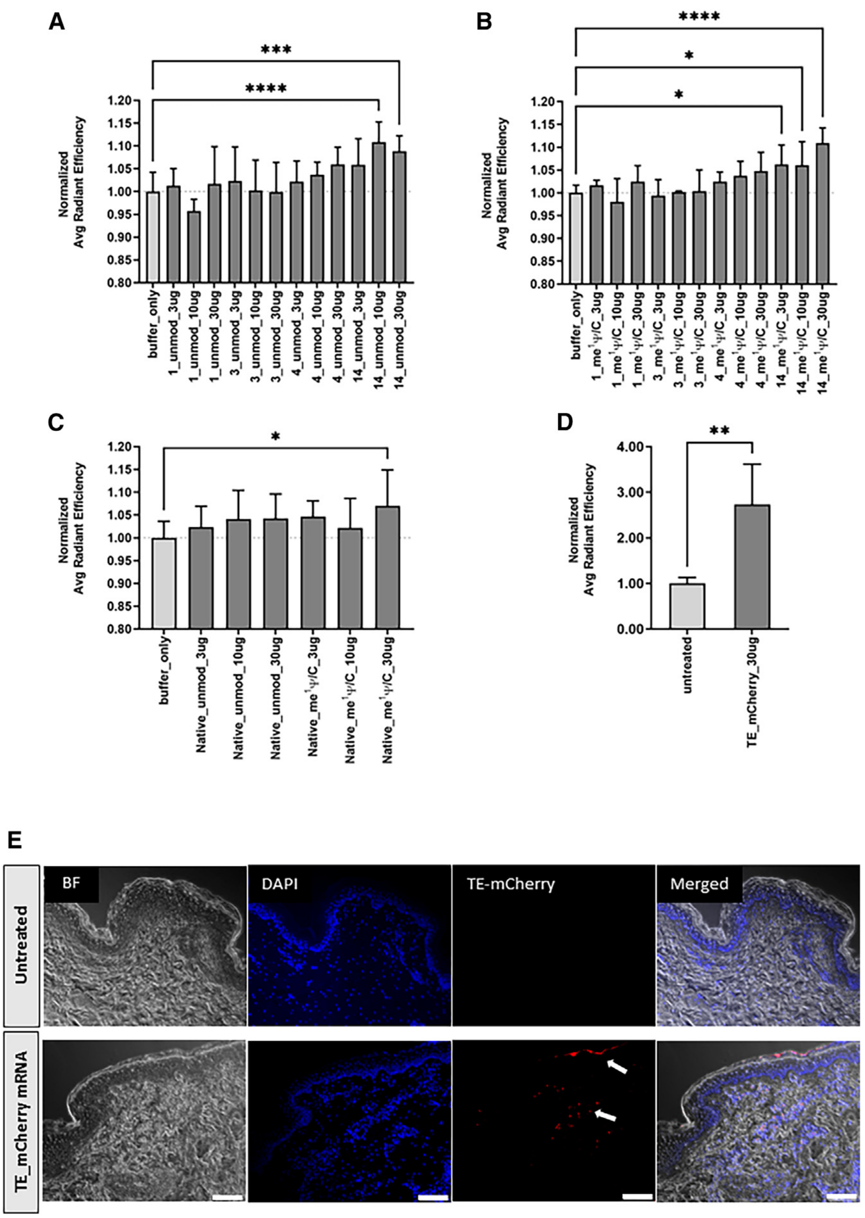

Figure 3. Analysis of elastin expression after *in vivo* intradermal delivery of TE mRNA variants into porcine skin using ElaNIR staining

Selected TE mRNA variants were formulated with Ringer's lactate (RL) buffer only. Using a BD Micro-Fine insulin syringe, 9 × 10 μL was injected into a defined skin area, which was marked with a tattoo ink pen. Only buffer without mRNA was injected as a control. Furthermore, at the end of the experiment, biopsies of untreated skin were collected. (A–C) Unmodified or $\text{me}^1\Psi$/C-modified TE mRNA variants 1, 3, 4, 14, and native were injected intradermally into pig skin in 90 μL RL containing 3, 10, or 30 μg of TE mRNA variants. Each mRNA was applied as a 5-fold replicate and tested in parallel in two pigs. After 48 h of application, the pigs were euthanized, and skin biopsies were stained with ElaNIR to detect the elastin content in the skin using IVIS. (D) Thirty micrograms of TE_mCherry mRNA in 90 μL RL was injected intradermally into porcine skin in quadruplicate or quintuplicate. After 48 h of application, the pigs were euthanized, skin biopsies were fixed in 4% PFA, and mCherry fluorescent signal was measured using IVIS. Fluorescence intensity was quantified as average radiant efficiency (p/s/cm^2/sr)/(μW/cm^2) and normalized to the corresponding buffer-only control. The results are shown as the mean + SD. Statistical differences were determined using one-way ANOVA followed Dunnett's multiple comparisons test (*$p < 0.05$, **$p < 0.01$, ***$p < 0.001$, ****$p < 0.0001$). (E) Microscopic fluorescence analyses of paraffin sections of porcine skin biopsies 48 h after intradermal application of 30 μg of TE_mCherry mRNA. Untreated skin biopsies served as negative controls. Arrows indicate the TE-mCherry produced in the skin. BF, bright field. DAPI, blue; mCherry, red. Scale bars: 100 μm.

reconstruction of elastic fibers in the elastase model of abdominal aortic aneurysm in rats, which reversed aneurysm dilatation.[31] Halm et al. investigated the long-term expression of a fluorescence-labeled human TE in human dermal fibroblasts.[32] Cells were transduced using a lentiviral vector to stably overexpress citrine-tagged TE, and the elastic fiber assembly was visualized for 7–14 days using confocal microscopy. In comparison to the use of viral vectors, the synthetic TE mRNA used in this study is directly translated into the desired protein in the cytoplasm; it does not need to enter the nucleus and therefore avoids a mutagenic risk.[2]

Compared with our previous study,[20] in which the native TE mRNA was modified with Ψ/m5C, in this study, replacement of the Ψ/m5C modification with me^1 Ψ/m5C or only me^1 Ψ in the native TE mRNA resulted in highly increased expression of TE protein *in vitro*. Furthermore, the codon-optimized TE_3 and TE_14 mRNA variants with Ψ/m5C modification resulted in significantly increased TE protein expression. Overall, the results demonstrated that the codon optimization of the TE mRNA influenced the TE protein production but had no significant influence on cell viability. In contrast, nucleotide modification had an influence on TE protein synthesis as well as cell viability. Thus, these results showed that, in addition to the CDS, nucleotide modification can also improve the protein expression efficiency in synthetic mRNA-based applications. Some codon optimizations even led to a decrease in protein expression, as seen in TE mRNA variants 1 and 4. This shows that not every codon optimization is successful and results in an improved protein expression.

Optimized codon sequences have been found to positively affect the translation and stability of the mRNA, leading to increased protein expression levels.[33] Thus, codon-optimized synthetic mRNAs have

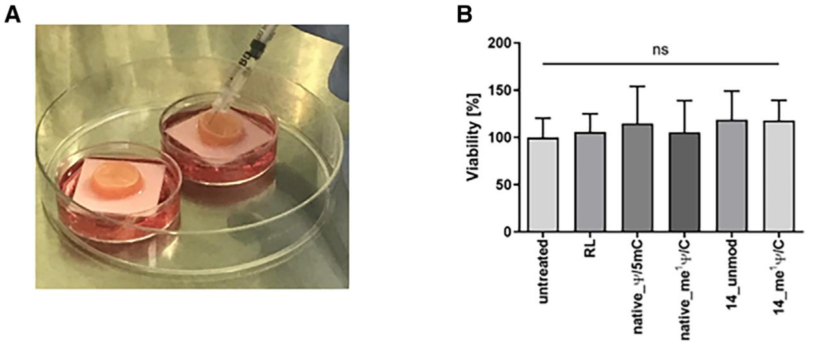

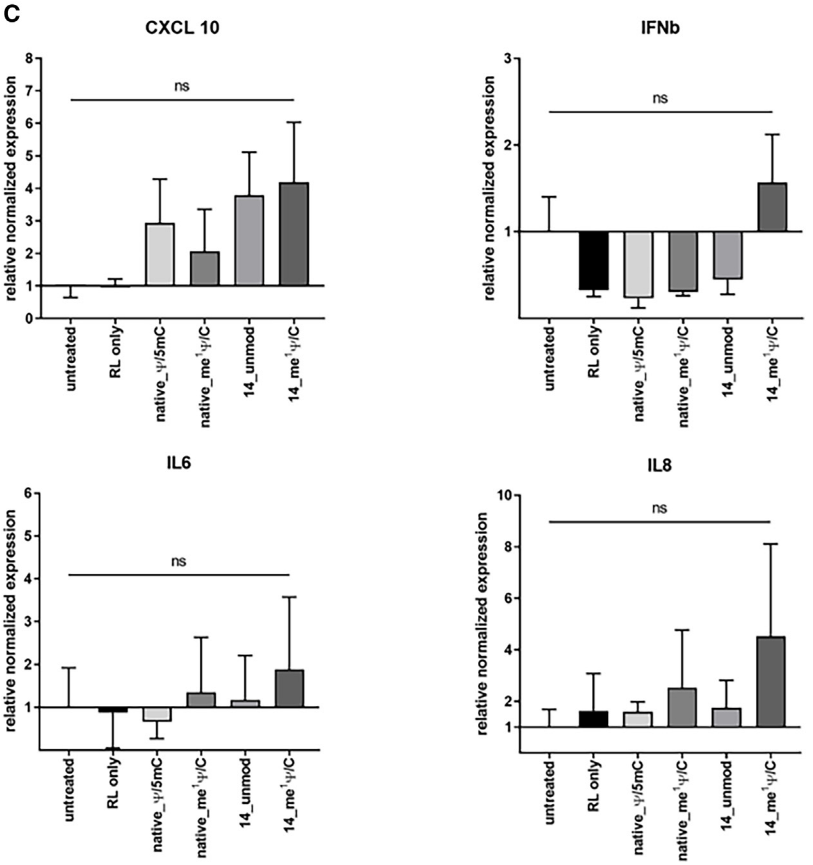

Figure 4. Analysis of cell viability and immune markers after injection of TE mRNA variants into human skin models

(A) Thirty micrograms of TE mRNA variants 14_me[1] Ψ/C, 14_unmod, native_me[1] Ψ/C, and native_Ψ/m5C in 90 μL RL (9 × 10 μL) was injected into human Phenion Full-Thickness (FT) skin models. Skin samples were incubated at 37°C and 5% CO_2 according to the manufacturer's instructions on filter paper in growth medium for 24 h. Only-RL-treated or untreated skin samples served as controls. (B) The cell viability in skin samples was determined 24 h after injection using MTT assay. The viability of the untreated skin samples was set to 100%. The results are shown as the mean + SEM (n = 3). (C) The immune activation was analyzed 24 h after injection of TE mRNA variants using qRT-PCR. RNA was isolated from untreated skin models or only-RL-buffer-injected skin models as controls. Gene expression levels were normalized to expression levels of the housekeeping gene glyceraldehyde 3-phosphate dehydrogenase (GAPDH) and presented as x-fold induction relative to the untreated skin model samples. The results are shown as the mean + SEM (n = 3). Statistical differences were determined using Friedman's test following Dunn's comparisons test.

been investigated in various studies and have shown successful expression of therapeutic proteins, such as IFN-α and erythropoietin (EPO) encoding mRNAs.[34,35] Karikó et al.[34] demonstrated enhanced translation of EPO mRNA constructs with specific AU- and GC-rich codons compared with the native sequence *in vitro*. In addition, substituting Ψ instead of U increased protein expression. *In vivo* studies in mice with optimized EPO mRNA confirmed the improved translational efficiency by an increase in EPO blood levels after a single administration of only 100 ng mRNA. By optimizing the native CDS, Hochmann et al. were able to significantly improve protein expression of IFN-α compared with mRNA constructs with native sequences. The efficiency was evaluated *in situ* in an explant of human skin tissue after biolistic delivery.[35] A significant increase in IFN-α protein expression was accomplished by exchanging the UTR and CDS modifications, varying the GC content, and maintaining a constant CAI. A comparison of these studies with our results confirms the importance of balanced CAI and GC content.

For TE mRNA, we demonstrated that a lower GC content has a positive impact on the protein expression efficiency of the mRNA. Increasing the CAI compared with native TE mRNA by choosing a lower GC content seems to be beneficial, as we observed for the TE_14 mRNA variant. However, although it was initially expected that the TE mRNA variant with the highest CAI should result in the highest protein expression, TE_14 mRNA with a slightly higher CAI than TE_native in combination with the nucleotide modification resulted in improved TE protein expression. In a very recent study, Nieuwkoop et al. investigated the correlations between codon usage and protein production in *E. coli* using machine-learning approaches.[36] Interestingly, the level of protein production could be predicted relatively accurately from the sequence information of the first eight codons, and it was found that, near the translation initiation site, the mRNA secondary structure rather than the CAI was the major determinant of protein production. Thus, in addition to the codon optimization, the newly generated secondary structures in the synthetic TE mRNA could also have an influence on protein translation. Furthermore, this powerful optimization tool should be used with caution, as some optimizations may lead

to the synthesis of novel peptides from alternative out-of-frame open reading frames (ORFs) and altered sites of post-transcriptional nucleotide modifications, resulting in the production of novel protein variants and ensembles.[37]

In our *in vivo* studies, 3, 10, and 30 μg of unmodified or me[1] Ψ/C-modified TE mRNA variants of native, 1, 3, 4, and 14 TE mRNA were injected in RL buffer into the skin of healthy young pigs. Compared with control skin sites, a significant increase in TE protein by 10 and 30 μg of the unmodified 14_TE mRNA variant was detected 48 h post-injection. An increase in TE amount of up to 10% was achieved by administration of the 14_TE mRNA variant with the me[1] Ψ modification. Here, a dose of only 3 μg was already sufficient for a significantly increased protein expression. When the native CDS was used, 30 μg was required to achieve an increase in TE expression. As in the *in vitro* studies, both the CDS and the use of me[1] Ψ had a major impact on the efficiency of protein expression. The nucleotide modification enabled a significantly increased protein translation to be reached at a lower dose than unmodified 14_TE mRNA. In the case of native TE mRNA, only the application of 30 μg and the me[1] Ψ modification resulted in significantly increased TE protein expression.

Karikó et al. set an important milestone in the optimization of synthetic mRNA by incorporating nucleobase modifications found in natural RNAs, such as Ψ, m5C, and m5U (5-methyluridine).[38] These nucleotides have been shown to stabilize the mRNA, resulting in highly increased protein expression by diminishing the activation of intrinsic and extrinsic RNA sensors such as TLRs and the protein kinase R (PKR), limiting activation of 2-5′-oligoadenylate synthetase and increasing mRNA resistance to cleavage by RNase L.[39] Similar to our study results, Andries et al. also demonstrated improved performance of me[1] Ψ modification alone and/or in combination with m5C compared with Ψ and/or Ψ/5mC modification in terms of improved protein expression *in vitro* and *in vivo* along with reduced immunogenicity.[40]

The use of me[1] Ψ was also utilized in the COVID-19 mRNA vaccines developed by both Pfizer-BioNTech and Moderna, which were the first approved mRNA therapeutics.[41] However, some studies have also shown that unmodified mRNA can be applied *in vivo*, suggesting that nucleotide modification may not be mandatory.[42] Our results indicate a similar situation: both unmodified and nucleotide-modified TE mRNA variants resulted in the expression of the target protein *in vivo*. Although strongly reduced protein expression and a high cell toxicity effect were observed after the transfection of unmodified TE mRNAs into cells, no increased cytotoxicity or immune activation was detected in the human skin model for the TE mRNA variants with the highest protein expression efficiency *in vivo*, 14_me[1] Ψ/C, 14_unmod, and native_ me[1] Ψ/C, as well as for native_Ψ/m5C mRNA. Thereby, using the maximum amount of 30 μg mRNA as in the *in vivo* studies, we confirmed that neither the unmodified mRNA nor the nucleotide-modified mRNA variants had cytotoxic effects. Furthermore, 48 h after the TE mRNA application into pig skin, no skin irritation or redness was visible at the injection sites. However, extensive analyses of the activation of the immune system and inflammation need to be performed in future long-term *in vivo* experiments with the selected TE mRNA variant.

Initial studies in mice and later in patients with type 2 diabetes mellitus showed that AZD8601, a modified mRNA encoding vascular endothelial growth factor (VEGF), improved wound healing in diabetic wound models in mice and increased skin blood flow in patients.[43] Similar to our studies, the mRNA modified with me[1] Ψ highly improved the tolerability of VEGF mRNA and increased the efficiency of protein expression *in vitro* as well as *in vivo*. In our study, we also demonstrated that local administration of the mRNA without a carrier was possible and led to the expression of the target protein. Other studies have also shown that the administration of a carrier-free mRNA, e.g., in RL buffer, is durable and results in successful expression of the target protein.[44,45] The possibility of carrier-free local delivery could also have a positive impact on the tolerability of the mRNA by circumventing the potentially toxic/irritating effects of the carrier itself.

After demonstrating the successful expression of TE protein, the major component (>90%) of elastin fibers,[30] both *in vitro* and *in vivo*, we next plan to perform long-term studies in *in vivo* models to assess elastin fiber formation, functionality, and longevity. In addition, extensive analyses of immune system activation and inflammation remain to be performed in future long-term *in vivo* experiments with the selected TE mRNA variant. In addition, it should be noted that the current method of administration by intradermal injection may allow local treatment of small areas of skin, such as scars. For the treatment of large skin areas or other organs/tissues, further development of novel delivery methods is needed, for example, targeted delivery after systemic application.

Conclusion

In conclusion, our study demonstrated that the codon optimization as well as nucleotide modification of synthetic TE mRNA resulted in a lead mRNA candidate, which led to an improved TE protein expression *in vitro* and *in vivo*. In addition to the well-known influences of nucleotide modifications on mRNA translation efficiency, toxicity, and stability, the codon optimization of the native mRNA sequence is another crucial factor. In this study, codon optimization of TE mRNA strongly affected the translation efficiency without influencing the cell viability *in vitro*, and nucleotide modifications of codon-optimized TE mRNA variants strongly modulated translation efficiency *in vitro* and reduced cellular toxicity. In future studies, we will investigate the regenerative and therapeutic potential of this newly designed auspicious TE mRNA variant for improving wound healing and preventing and restoring scar tissue, as well as for other diseases, such as myocardial infarction or aneurysms, that require *de novo* elastin synthesis.

MATERIALS AND METHODS

In vitro synthesis of TE mRNA variants

The CDS of the TE mRNA variants was selected considering the GC content and the CAI, which defines the relative adaptiveness of the

codon usage of a gene toward the codon usage of highly expressed genes.[46] Higher values indicate a higher proportion of the most abundant codons, which in this case fits optimally to the human translation machinery, leading to higher protein expression levels. Four differentially modified TE mRNA variants were compared with native human TE mRNA for their protein translation efficacy. In total, five TE sequence candidates were determined for *in vitro* analysis (Table 1).

The synthesis of TE mRNA variants was performed by IVT as described in our previous study.[20] TE-encoding DNA was amplified using pcDNA 3.3 or pUC57 plasmids containing different codon-optimized sequences for human TE. The plasmids were produced by Aldevron (Fargo, ND, USA). PCR was performed using the HotStar HiFidelity Polymerase Kit (Qiagen, Hilden, Germany) together with 0.7 mM each forward (5′-TTGGACCCTCGTACA GAAGCTAATACG-3′) and reverse primers (5′-T_{120}-CTTCCTACT CAGGCTTTATTCAAAGACCA-3′) to amplify the plasmid insert. During the amplification, a poly(T)-tail of 120 thymidines (T) was added to the plasmid insert. Primers were purchased from ELLA Biotech (Martinsried, Germany). The following cycling protocol was used for the PCR: initial activation at 94°C for 3 min and 30 cycles of denaturation at 94°C for 45 s, annealing at 60°C for 1 min, and extension at 72°C for 1 min. After the final extension at 72°C for 5 min, the amplified PCR products were purified using the QIAquick PCR purification kit (Qiagen, Hilden, Germany) according to the manufacturer's instructions.

Afterward, 1.5 μg of each PCR product was *in vitro* transcribed using the MEGAscript T7 Kit (Life Technologies, Darmstadt, Germany) according to the manufacturer's instructions. Different mRNA variants were generated (Table 2). To produce unmodified mRNA variants, 1.875 mM GTP, 7.5 mM ATP, 7.5 mM CTP, and UTP were used. Modified mRNA variants were generated by using 7.5 mM Ψ or me^1 Ψ instead of UTP, and instead of CTP, 7.5 mM m5CTP (m5C) was used. CTP and UTP were used from the MEGAscript T7 Kit and the other nucleotides were purchased from TriLink BioTechnologies (San Diego, CA, USA). To each IVT reaction, 2.5 mM 3′-O-Me-m7G(5′)ppp(5′)G RNA cap structure analog (New England Biolabs, Frankfurt am Main, Germany) and 40 U RiboLock RNase inhibitor (Thermo Scientific, Waltham, MA, USA) were added. After an incubation of 4 h at 37°C, 1 μL of TurboDNase was added to remove the DNA template. After further incubation for 15 min at 37°C, the mRNA was purified using the RNeasy Mini Kit (Qiagen, Hilden, Germany) according to the manufacturer's instructions and dephosphorylated at 37°C for 30 min using 15 U Antarctic phosphatase (New England Biolabs, Frankfurt am Main, Germany). Subsequently, the mRNA was purified using the RNeasy Mini Kit. The concentrations of DNA and mRNA products were determined using BioPhotometer (Eppendorf, Hamburg, Germany). The purity and quality of amplified DNA and synthetic mRNA were analyzed using 1% agarose gel electrophoresis (1 h, 100 V) and subsequent staining with GelRed (Biotium, Fremont, CA, USA) in 1× Tris-borate-EDTA (TBE) buffer.

Cultivation of cells

EA.hy926 cells (ATCC, Manassas, VA, USA) were cultivated in Dulbecco's modified Eagle's medium (DMEM) with high glucose and L-glutamine containing 10% heat-inactivated fetal bovine serum (FBS) at 37°C and 5% CO_2. Upon reaching 80% confluency, cells were passaged. Thereafter, the cells were washed with Dulbecco's phosphate-buffered saline (DPBS) and detached using 0.05% trypsin-EDTA. The cell culture medium was changed every 3–4 days. All cell culture reagents were obtained from Fisher Scientific.

Transfection of cells with TE mRNA

To perform the transfection of EA.hy926 cells, 3×10^5 cells were seeded in 2 mL cell medium in each well of a six-well plate and cultivated at 37°C and 5% CO_2 for 24 h. Lipoplexes were generated by complexing 2.5 μg TE mRNA with 4 μL L2000 in 1 mL OptiMEM I reduced serum-free medium for 20 min at room temperature. Cells were washed 1× with DPBS and incubated for 4 h with the lipoplexes at 37°C and 5% CO_2. The transfection medium was then replaced with 1 mL cell culture medium, and the cells were incubated at 37°C and 5% CO_2 for 24 to 72 h. As controls, cells were also incubated only with OptiMEM (medium) or OptiMEM with 4 μL L2000. All reagents were obtained from Thermo Fisher Scientific. After 24 and 72 h of cultivation, TE protein expression in the supernatant was detected using ELISA.

Elastin/TE ELISA

Supernatants were collected 24, 48, and 72 h after transfection and centrifuged for 10 min at 3,000*g* at room temperature. Afterward, 750 μL supernatant was transferred into a new protein low-binding tube, snap-frozen in liquid nitrogen, and stored at −80°C until the analysis was performed. TE concentration in the collected supernatants was determined using the ELISA kit for human elastin (Biozol, Cloud-Clone, Eching, Germany) according to the manufacturer's instructions. Supernatants of cells transfected with TE mRNA variants containing modified nucleotides were diluted 1:50 with DBPS. All other supernatants were used undiluted.

Detection of cell viability by PrestoBlue assay

The impact of different mRNA variants on cell viability was analyzed via PrestoBlue assay. Thereby, 3×10^5 EA.hy926 cells were seeded in a six-well plate, cultivated for 24 h at 37°C and 5% CO_2, and transfected with 2.5 μg TE mRNA complexed with 4 μL of L2000 in OptiMEM for 4 h at 37°C and 5% CO_2. The transfection medium was replaced with cell culture medium after 4 h, and the cells were incubated at 37°C and 5% CO_2 for 24 h. Cells treated with either L2000 or OptiMEM (medium) served as controls. After 24 h, the cells were washed 1× with DPBS, and 500 μL of 1:10 diluted PrestoBlue working solution (Invitrogen, Carlsbad, CA, USA) in cell culture medium was added to each well and incubated for 1.5 h at 37°C. Using a multimode microplate reader (Mithras LB 940; Berthold Technologies), 100 μL of each sample was measured in triplicate with 530 nm excitation and 600 nm emission wavelengths.

Analysis of the presence of TE mRNA in the cells

The amount of TE mRNA in EA.hy926 cells was detected 48 and 72 h after the transfection of cells with 2.5 μg TE mRNA.

Isolation of RNA and cDNA synthesis

Cells were washed with 1 mL DPBS, detached with 0.05% trypsin-EDTA, and centrifuged at 1,000*g* for 5 min at room temperature. Subsequently, the cells were washed 1× with DPBS and centrifuged for 5 min at 1,000*g* at room temperature. The cell pellets were snap-frozen in liquid nitrogen and stored at −80°C until the detection of synthetic TE mRNA in the cells. RNA was isolated using a standard Trizol protocol. Briefly, 1 mL Trizol (Invitrogen, Carlsbad, CA, USA) was added to the frozen cell pellet and vortexed until the cells were completely lysed. Then, 0.2 mL chloroform was added and centrifuged at 12,000*g* for 15 min at 2°C–8°C. The aqueous phase was transferred to a new tube, and 0.5 mL of 2-propanol was added. After mixing and incubation for 15 min at room temperature, the mixture was centrifuged at 12,000*g* for 10 min at 2°C–8°C. The RNA precipitate formed a pellet that was washed with 95% EtOH and dissolved in RNase-free water after drying.

To synthesize cDNA, 1 μg of RNA was transcribed using the iScript cDNA Synthesis Kit (Bio-Rad, Hercules, CA, USA), according to the manufacturer's instructions, and then stored at −20°C until use.

Real-time quantitative reverse transcription-polymerase chain reaction (qRT-PCR)

To determine the amount of TE mRNA by qRT-PCR, cDNA standards with known TE mRNA content were used to generate a standard curve. Standard curves were generated starting at 3 ng using a 100-fold serial dilution series of four template concentrations, and all reactions were performed in duplicate. After the detection of the target in each standard sample, the standard curve was plotted as Cq versus the logarithm of the template concentration. The concentration of TE mRNA in the samples was within the concentration range covered by the standard curve, and the quantities of TE mRNA in cell pellets or skin biopsies were determined using the standard curve. The quantities were shown as nanograms TE mRNA/total RNA used for cDNA synthesis.

In vivo studies in pigs

Ethics statement

The study was performed in accordance with the Federation of European Laboratory Animal Science Associations (FELASA) and the American Association for Laboratory Animal Science (AALAS) recommendations for the care and use of laboratory animals. Experiments were approved by the institutional animal care committee and review board and conformed to Austrian law (BMBWF-68.205/0088-V/3b/2019).

Animals and experimental setup

Six 12-week-old domestic pigs (*Sus scrofa domestica*) weighing approximately 30 kg were obtained from a local, specific-pathogen-free breeding facility (Gutshof Medau/Schweineanlage, A-2560 Berndorf). The animal experiments were performed at the University of Veterinary Medicine Vienna and the animals were housed in the stables of the University Hospital for pigs. After a 1-week acclimation period, the experiments started and lasted 48 h. Animals were clinically examined daily 48 h before the start of the experiment until the end of the experiment.

A total of six pigs were used to test 11 mRNA variants. Each mRNA was administered in parallel in two pigs in triplicate per pig. Application of the different mRNA variants with different nucleotide modifications, marking of the application sites with a permanent marker, and euthanasia were performed under anesthesia by intramuscular injection of ketamine hydrochloride (Narketan, 10 mg/kg body weight) and azaperone (Stresnil, 1.3 mg/kg body weight). The sampling of the marked biopsies was performed postmortem after intracardiac injection of T61 (1 mL/10 kg body weight).

In vivo *application of TE mRNA*

All unmodified and $me^1\Psi$/C TE mRNA variants were evaluated for their *in vivo* TE protein expression efficiency after intradermal application in porcine skin. Furthermore, to identify the newly produced exogenously expressed TE protein in the skin, a TE variant expressing a mCherry-tagged version of TE (TE_mCherry) was also injected. Each mRNA was dissolved in RL buffer (Fresenius Kabi, Graz, Austria) in a total volume of 90 μL at concentrations of 3, 10, and 30 μg. For the application of TE_mCherry mRNA, 30 μg was used. Intradermal injections were performed using the insulin syringe BD Micro-Fine (BD, Franklin Lakes, NJ, USA) and injecting 9 × 10 μL into a defined skin area of 1 × 1 cm. As a control, only RL buffer without mRNA was injected. Animals were euthanized 48 h after injection, and all injection sites were biopsied using a 10 mm biopsy punch. Furthermore, biopsies of untreated skin were taken at the end of the experiment. The biopsies were snap-frozen in liquid nitrogen and stored at −80°C until analysis of elastin content using elastin-specific ElaNIR staining.

Staining of skin biopsies with ElaNIR and detection

The fluorescent dye ElaNIR[47] was used to detect elastin in the skin. Therefore, 1 μmol ElaNIR was dissolved in 1 mL DMSO (Sigma-Aldrich, St. Louis, MO, USA), and skin biopsies were incubated with 750 μL DPBS containing 10% DMSO and 20 μM ElaNIR at 4°C overnight and then washed six times with DPBS for 30 min at room temperature. The near-infrared fluorescence signal (excitation 745 nm, emission 800 nm) was detected using an IVIS (IVIS Spectrum, PerkinElmer). Images were analyzed with the Living Image version 4.4 software (PerkinElmer). The fluorescence intensity in defined regions of interest (ROIs) was quantified as the average radiant efficiency ($p/s/cm^2/sr$)/($\mu W/cm^2$) after subtracting the background signal. Data were normalized to corresponding controls.

Histological analysis of skin biopsies injected with TE_mCherry mRNA

Skin biopsies were collected, stored in 70% ethanol (PanReac AppliChem ITW Reagents, Darmstadt, Germany), transferred to

embedding cassettes, and fixed in 4% paraformaldehyde (PFA; Merck, Darmstadt, Germany) overnight at 4°C. Samples were then dehydrated and infiltrated with paraffin in an automatic tissue processor and embedded into paraffin blocks using a tissue-embedding machine. Blocks were cut into 5-μm-thick sections using a microtome (Thermo Fischer Scientific), mounted on SuperFrost microscope slides (R. Langenbrinck, Emmendingen, Germany), and dried overnight at room temperature in the dark. The paraffin sections were deparaffinized twice for 2 min in 100% xylene (PanReac AppliChem ITW Reagents, Darmstadt, Germany) and then rehydrated using a graded ethanol series (100%, 80%, 70%, 60%) for 2 min each and washed for 1 min in distilled, deionized water. Staining of cell nuclei was performed using Vectashield mounting medium (Vector Laboratories, Burlingame, CA, USA) containing the fluorescent dye DAPI. Fluorescence images were acquired using the Axiovert135 fluorescence microscope (Zeiss) and analyzed using AxioVision Rel 4.8 software.

Analysis of cytotoxicity and immune activation potential in human skin model

Application of TE mRNA in human skin model

Using the human Phenion FT skin model (Henkel, Düsseldorf, Germany), the potentially toxic and immunogenic effects of synthetic TE mRNA variants were analyzed after intradermal application. All components for the cultivation of the FT skin model were purchased from Henkel. The skin models were placed in the air-liquid interphase culture system in a Petri dish and incubated with an air-liquid interface (ALI) medium at 37°C and 5% CO_2 for 24 h. Then, 90 μL RL buffer without or with 30 μg TE mRNA variants 14_me^1 Ψ/C, 14_unmod, native_me^1 Ψ/C, or native_Ψ/m5C was injected (9 × 10 μL) into the FT skin model using the insulin syringe BD Micro-Fine. The skin models were cultivated for a further 24 h at 37°C and 5% CO_2. FT skin models that were untreated or injected with RL buffer only served as controls. For each treatment, six skin models were used, with three skin models used for immune activation analysis and three skin models used for cytotoxicity analysis.

In vitro *skin toxicity analysis*

Skin toxicity was analyzed after the injection of TE mRNA variants and control groups into FT skin models using the MTT assay according to OECD 439 guidelines. All skin samples were washed eight times with 600 μL DPBS 24 h after injection and incubated for 3 h per well of a 24-well plate filled with 1 mL DPBS containing 0.5 mg/mL MTT working solution (Sigma, St. Louis, MO, USA) at 37°C and 5% CO_2. Skin models were then dried, transferred into a 24-well plate containing 1 mL 2-propanol per well (VWR International, Radnor, PA, USA), and incubated overnight with shaking at 4°C to elute formazan from the skin models. Skin models were removed from the wells, and the eluted formazan was diluted 1:1 with 1 mL 2-propanol. From each sample, 200 μL was transferred per well of a 96-well plate, and the absorbance was measured at 540 nm using a microplate reader (Mithras, Bad Wildbach, Germany).

RNA isolation from skin model

RNA isolation was performed 24 h after injection of mRNA into the Phenion FT skin model using the RNeasy Mini Kit (Qiagen, Hilden, Germany). Half of the skin tissue was cut into eight pieces and transferred into 350 μL RTL buffer supplemented with 10 μL β-mercaptoethanol/mL buffer (Sigma, St. Louis, MO, USA) and incubated at 300 rpm for 35 min at room temperature in a thermomixer. The tissue homogenate was then mixed with 500 μL of RNase-free water and 10 μL proteinase K (both from Qiagen), incubated at 55°C for 40 min, and centrifuged at 8,000*g* for 30 s. The supernatant was collected in a new reaction tube and gently mixed with 0.5 volumes of 100% ethanol. Then, 700 μL was transferred into the RNeasy Mini Kit spin column and centrifuged at 8,000*g* for 15 s, and the flowthrough was discarded. The remaining tissue lysate was added to the spin column and the procedure was repeated. The columns were washed with 350 μL RW1 buffer and centrifuged for 15 s at 8,000 *g*. To remove DNA, 80 μL of DNase from the RNase-free DNase Set (Qiagen) was prepared according to the manufacturer's instructions, added to each column, and incubated for 15 min. Next, 350 μL RW1 buffer was added and the columns were centrifuged at 8,000*g* for 15 s. After the flowthrough was discarded, the columns were washed twice with 500 μL RPE buffer and centrifuged at 8,000*g* for 30 s. The buffer was removed and columns were centrifuged at maximal speed for 4 min. RNA was eluted with 50 μL RNase-free water and centrifuged at 8,000 *g* for 1 min. The isolated RNA was snap-frozen in liquid nitrogen and stored at −80°C.

qRT-PCR

The expression of immune activation markers IL-6, IL-8, CXCL10, and IFN-β in the *in vitro* human skin model was investigated by qRT-PCR. Total RNA was isolated as described earlier and cDNA synthesis was performed using 900 ng of isolated RNA and iScript cDNA Synthesis Kit (Bio-Rad) under the following conditions: 5 min at 25°C, 30 min at 42°C, and 5 min at 85°C. The qRT-PCR was performed with 1:10 diluted cDNA and the iQ SYBR Green Supermix (Bio-Rad) according to the manufacturer's instructions. The reactions were run in triplicate in an iCycler iQ real-time PCR detection system (Bio-Rad). Primers[48] used for the specific amplification of transcripts were purchased from Ella Biotech (Martinsried, Germany). The expression of glyceraldehyde 3-phosphate dehydrogenase (GAPDH) served as an internal control and was used to normalize expression levels. The results are shown relative to control mRNA levels in untreated samples.

Statistics

Data are shown as means ± SEM. Statistical analysis of data was performed using GraphPad Prism version 9.0.1. One-way ANOVA for repeated measurements and Bonferroni's or Tukey's multiple comparison or Friedmann's test following Dunn's comparisons test were applied. $p < 0.05$ was considered statistically significant.

DATA AND CODE AVAILABILITY

The authors confirm that the data supporting the findings of this study are available within the article and supplemental information.

Raw data supporting the findings of this study are available from the corresponding author upon reasonable request.

SUPPLEMENTAL INFORMATION

Supplemental information can be found online at https://doi.org/10.1016/j.omtn.2023.07.035.

ACKNOWLEDGMENTS

We thank Prof. Peter Pilz (Department of Animal Physiology, University of Tübingen) for his assistance in the statistical analysis of the data. This project has received funding from the Eurostars-2 joint program with cofunding from the European Union Horizon 2020 research and innovation program (01QE1839C/E! 12338 RELAST). Furthermore, we acknowledge the support of the Open Access Publishing Fund of the University of Tübingen.

AUTHOR CONTRIBUTIONS

S.G., T.H., A.S., M.M., and M.A.-A. planned and designed the experiments. S.G and T.H. performed experiments with support from L.H., J.W., S.S., and R.M. A.L. and C.K. supported all *in vivo* experiments. S.G. and T.H. analyzed the data. Y.-T.C. provided ElaNIR dye and supported the elastin staining experiments. R.M. supported the performance of IVIS and the analyses of the data. J.L. performed histological sections. H.P.W., M.A.-A., and C.S. contributed reagents/materials/analysis tools. S.G. and M.A.-A. wrote the manuscript and prepared the figures. M.A.-A. supervised the project and revised the manuscript.

DECLARATION OF INTERESTS

The authors have no competing interests to declare.

REFERENCES

1. Steinle, H., Weber, J., Stoppelkamp, S., Große-Berkenbusch, K., Golombek, S., Weber, M., Canak-Ipek, T., Trenz, S.M., Schlensak, C., and Avci-Adali, M. (2021). Delivery of synthetic mRNAs for tissue regeneration. Adv. Drug Deliv. Rev. *179*, 114007.
2. Sahin, U., Karikó, K., and Türeci, Ö. (2014). mRNA-based therapeutics–developing a new class of drugs. Nat. Rev. Drug Discov. *13*, 759–780.
3. Avci-Adali, M., Behring, A., Steinle, H., Keller, T., Krajeweski, S., Schlensak, C., and Wendel, H.P. (2014). In vitro synthesis of modified mRNA for induction of protein expression in human cells. J. Vis. Exp. e51943.
4. Yamamoto, A., Kormann, M., Rosenecker, J., and Rudolph, C. (2009). Current prospects for mRNA gene delivery. Eur. J. Pharm. Biopharm. *71*, 484–489.
5. Uitto, J. (1979). Biochemistry of the elastic fibers in normal connective tissues and its alterations in diseases. J. Invest. Dermatol. *72*, 1–10.
6. Baldwin, A.K., Simpson, A., Steer, R., Cain, S.A., and Kielty, C.M. (2013). Elastic fibres in health and disease. Expet Rev. Mol. Med. *15*, e8.
7. Shapiro, S.D., Endicott, S.K., Province, M.A., Pierce, J.A., and Campbell, E.J. (1991). Marked longevity of human lung parenchymal elastic fibers deduced from prevalence of D-aspartate and nuclear weapons-related radiocarbon. J. Clin. Invest. *87*, 1828–1834.
8. Moore, J., and Thibeault, S. (2012). Insights Into the Role of Elastin in Vocal Fold Health and Disease. J. Voice *26*, 269–275.
9. Chin, K., Wieslander, C., Shi, H., Balgobin, S., Montoya, T.I., Yanagisawa, H., and Word, R.A. (2016). Pelvic Organ Support in Animals with Partial Loss of Fibulin-5 in the Vaginal Wall. PLoS One *11*, e0152793.
10. Wang, K., Meng, X., and Guo, Z. (2021). Elastin structure, synthesis, regulatory mechanism and relationship with cardiovascular diseases. Front. Cell Dev. Biol. *9*, 596702.
11. Schmelzer, C.E.H., and Duca, L. (2022). Elastic fibers: formation, function, and fate during aging and disease. FEBS J. *289*, 3704–3730.
12. Ma, J., Ma, C., Li, J., Sun, Y., Ye, F., Liu, K., and Zhang, H. (2020). Extracellular Matrix Proteins Involved in Alzheimer's Disease. Chemistry *26*, 12101–12110.
13. Szychowski, K.A., Skóra, B., and Wójtowicz, A.K. (2022). Elastin-Derived Peptides in the Central Nervous System: Friend or Foe. Cell. Mol. Neurobiol. *42*, 2473–2487.
14. Heinz, A. (2021). Elastic fibers during aging and disease. Ageing Res. Rev. *66*, 101255.
15. Ozsvar, J., Yang, C., Cain, S.A., Baldock, C., Tarakanova, A., and Weiss, A.S. (2021). Tropoelastin and Elastin Assembly. Front. Bioeng. Biotechnol. *9*, 643110.
16. Kumari, S., Panda, T.K., and Pradhan, T. (2017). Lysyl Oxidase: Its Diversity in Health and Diseases. Indian J. Clin. Biochem. *32*, 134–141.
17. Duque Lasio, M.L., and Kozel, B.A. (2018). Elastin-driven genetic diseases. Matrix Biol. *71–72*, 144–160.
18. Rnjak, J., Wise, S.G., Mithieux, S.M., and Weiss, A.S. (2011). Severe burn injuries and the role of elastin in the design of dermal substitutes. Tissue Eng. B Rev. *17*, 81–91.
19. Hafemann, B., Ensslen, S., Erdmann, C., Niedballa, R., Zühlke, A., Ghofrani, K., and Kirkpatrick, C.J. (1999). Use of a collagen/elastin-membrane for the tissue engineering of dermis. Burns *25*, 373–384.
20. Lescan, M., Perl, R.M., Golombek, S., Pilz, M., Hann, L., Yasmin, M., Behring, A., Keller, T., Nolte, A., Gruhn, F., et al. (2018). De Novo Synthesis of Elastin by Exogenous Delivery of Synthetic Modified mRNA into Skin and Elastin-Deficient Cells. Mol. Ther. Nucleic Acids *11*, 475–484.
21. Hussain, S.H., Limthongkul, B., and Humphreys, T.R. (2013). The biomechanical properties of the skin. Dermatol. Surg. *39*, 193–203.
22. Wise, S.G., and Weiss, A.S. (2009). Tropoelastin. Int. J. Biochem. Cell Biol. *41*, 494–497.
23. Baumann, L., Bernstein, E.F., Weiss, A.S., Bates, D., Humphrey, S., Silberberg, M., and Daniels, R. (2021). Clinical Relevance of Elastin in the Structure and Function of Skin. Aesthet. Surg. J. Open Forum *3*, ojab019.
24. Kucich, U., Rosenbloom, J.C., Abrams, W.R., and Rosenbloom, J. (2002). Transforming growth factor-beta stabilizes elastin mRNA by a pathway requiring active Smads, protein kinase C-delta, and p38. Am. J. Respir. Cell Mol. Biol. *26*, 183–188.
25. Sommer, N., Sattler, M., Weise, J.M., Wenck, H., Gallinat, S., and Fischer, F. (2013). A tissue-engineered human dermal construct utilizing fibroblasts and transforming growth factor beta1 to promote elastogenesis. Biotechnol. J. *8*, 317–326.
26. Rich, C.B., Ewton, D.Z., Martin, B.M., Florini, J.R., Bashir, M., Rosenbloom, J., and Foster, J.A. (1992). IGF-I regulation of elastogenesis: comparison of aortic and lung cells. Am. J. Physiol. *263*, L276–L282.
27. Rothuizen, T.C., Kemp, R., Duijs, J.M.G.J., de Boer, H.C., Bijkerk, R., van der Veer, E.P., Moroni, L., van Zonneveld, A.J., Weiss, A.S., Rabelink, T.J., and Rotmans, J.I. (2016). Promoting Tropoelastin Expression in Arterial and Venous Vascular Smooth Muscle Cells and Fibroblasts for Vascular Tissue Engineering. Tissue Eng. C Methods *22*, 923–931.
28. Akhurst, R.J., and Derynck, R. (2001). TGF-β signaling in cancer—a double-edged sword. Trends Cell Biol. *11*, S44–S51.
29. Mithieux, S.M., and Weiss, A.S. (2017). Design of an elastin-layered dermal regeneration template. Acta Biomater. *52*, 33–40.
30. Mitzmacher, M.G., Mithieux, S.M., Weiss, A.S., Hee, C.K., and Daniels, R. (2020). Novel Recombinant Tropoelastin Implants Restore Skin Extracellular Matrix. J. Drugs Dermatol. JDD *19*, 1166–1172.
31. Xiong, J., Wang, S.M., Chen, L.H., Lin, Y., Zhu, Y.F., and Ye, C.S. (2008). Elastic fibers reconstructed using adenovirus-mediated expression of tropoelastin and tested in the elastase model of abdominal aortic aneurysm in rats. J. Vasc. Surg. *48*, 965–973.
32. Halm, M., Schenke-Layland, K., Jaspers, S., Wenck, H., and Fischer, F. (2016). Visualizing tropoelastin in a long-term human elastic fibre cell culture model. Sci. Rep. *6*, 20378.

33. Presnyak, V., Alhusaini, N., Chen, Y.H., Martin, S., Morris, N., Kline, N., Olson, S., Weinberg, D., Baker, K.E., Graveley, B.R., and Coller, J. (2015). Codon optimality is a major determinant of mRNA stability. Cell *160*, 1111–1124.

34. Karikó, K., Muramatsu, H., Keller, J.M., and Weissman, D. (2012). Increased erythropoiesis in mice injected with submicrogram quantities of pseudouridine-containing mRNA encoding erythropoietin. Mol. Ther. *20*, 948–953.

35. Hochmann, S., Mittermeir, M., Santic, R., Koszik, F., Griessner, L., Sonderegger, A.S., Hoffmann, T., Russe, E., Scheiblhofer, S., Weiss, R., et al. (2018). Evaluation of modified Interferon alpha mRNA constructs for the treatment of non-melanoma skin cancer. Sci. Rep. *8*, 12954.

36. Nieuwkoop, T., Terlouw, B.R., Stevens, K.G., Scheltema, R.A., De Ridder, D., Van der Oost, J., and Claassens, N.J. (2023). Revealing determinants of translation efficiency via whole-gene codon randomization and machine learning. Nucleic Acids Res. *51*, 2363–2376.

37. Mauro, V.P., and Chappell, S.A. (2014). A critical analysis of codon optimization in human therapeutics. Trends Mol. Med. *20*, 604–613.

38. Karikó, K., Buckstein, M., Ni, H., and Weissman, D. (2005). Suppression of RNA recognition by Toll-like receptors: the impact of nucleoside modification and the evolutionary origin of RNA. Immunity *23*, 165–175.

39. Anderson, B.R., Muramatsu, H., Jha, B.K., Silverman, R.H., Weissman, D., and Karikó, K. (2011). Nucleoside modifications in RNA limit activation of 2'-5'-oligoadenylate synthetase and increase resistance to cleavage by RNase L. Nucleic Acids Res. *39*, 9329–9338.

40. Andries, O., Mc Cafferty, S., De Smedt, S.C., Weiss, R., Sanders, N.N., and Kitada, T. (2015). N(1)-methylpseudouridine-incorporated mRNA outperforms pseudouridine-incorporated mRNA by providing enhanced protein expression and reduced immunogenicity in mammalian cell lines and mice. J. Contr. Release *217*, 337–344.

41. Pardi, N., Hogan, M.J., and Weissman, D. (2020). Recent advances in mRNA vaccine technology. Curr. Opin. Immunol. *65*, 14–20.

42. Kauffman, K.J., Mir, F.F., Jhunjhunwala, S., Kaczmarek, J.C., Hurtado, J.E., Yang, J.H., Webber, M.J., Kowalski, P.S., Heartlein, M.W., DeRosa, F., and Anderson, D.G. (2016). Efficacy and immunogenicity of unmodified and pseudouridine-modified mRNA delivered systemically with lipid nanoparticles in vivo. Biomaterials *109*, 78–87.

43. Gan, L.M., Lagerström-Fermér, M., Carlsson, L.G., Arfvidsson, C., Egnell, A.C., Rudvik, A., Kjaer, M., Collén, A., Thompson, J.D., Joyal, J., et al. (2019). Intradermal delivery of modified mRNA encoding VEGF-A in patients with type 2 diabetes. Nat. Commun. *10*, 871.

44. Phua, K.K.L., Leong, K.W., and Nair, S.K. (2013). Transfection efficiency and transgene expression kinetics of mRNA delivered in naked and nanoparticle format. J. Contr. Release *166*, 227–233.

45. Probst, J., Weide, B., Scheel, B., Pichler, B.J., Hoerr, I., Rammensee, H.G., and Pascolo, S. (2007). Spontaneous cellular uptake of exogenous messenger RNA in vivo is nucleic acid-specific, saturable and ion dependent. Gene Ther. *14*, 1175–1180.

46. Sharp, P.M., and Li, W.H. (1987). The codon Adaptation Index–a measure of directional synonymous codon usage bias, and its potential applications. Nucleic Acids Res. *15*, 1281–1295.

47. Su, D., Teoh, C.L., Park, S.-J., Kim, J.-J., Samanta, A., Bi, R., Dinish, U., Olivo, M., Piantino, M., Louis, F., et al. (2018). Seeing Elastin: A Near-Infrared Zwitterionic Fluorescent Probe for In Vivo Elastin Imaging. Chem *4*, 1128–1138.

48. Avci-Adali, M., Hann, L., Michel, T., Steinle, H., Stoppelkamp, S., Stang, K., Narita, M., Schlensak, C., and Wendel, H.P. (2015). In vitro test system for evaluation of immune activation potential of new single-stranded DNA-based therapeutics. Drug Test. Anal. *7*, 300–308.

OMTN, Volume *33*

Supplemental information

Improved tropoelastin synthesis in the skin by codon optimization and nucleotide modification of tropoelastin-encoding synthetic mRNA

Sonia Golombek, Thomas Hoffmann, Ludmilla Hann, Markus Mandler, Sabine Schmidhuber, Josefin Weber, Young-Tae Chang, Roman Mehling, Andrea Ladinig, Christian Knecht, Johanna Leyens, Christian Schlensak, Hans Peter Wendel, Achim Schneeberger, and Meltem Avci-Adali

SUPPLEMENTAL DATA

Table S1: Sequences of TE coding sequences

Table S2: Ranking list of in vitro tested TE mRNA variants.

Ranking	TE mRNA variant	Elastin [ng/mL]	Viability [%]
**1**	14_me1Ψ/m5C	7381	71.66
**2**	14_me1Ψ/C	6522	61.37
**3**	3_me1Ψ/m5C	5540	70.08
**4**	native_me1Ψ/C	4548	57.84
**5**	native_me1Ψ/m5C	2566	62.86
**6**	4_me1Ψ/C	2563	60.43
**7**	1_me1Ψ/C	2127	69.97
**8**	3_me1Ψ/C	2021	58.33
**9**	14_Ψ/m5C	1106	42.44
**10**	3_Ψ/m5C	887.4	40.75
**11**	native_Ψ/m5C	324.2	42.97
**12**	4_me1Ψ/m5C	216.9	79.18
**13**	native_unmod	33.84	1.60
**14**	3_unmod	29.05	1.1
**15**	4_unmod	28.54	1.59
**16**	1_unmod	26.36	3.01
**17**	4_Ψ/m5C	22.09	50.28
**18**	14_unmod	20.75	1.41
**19**	1_me1Ψ/m5C	15.25	77.24
**20**	1_Ψ/m5C	0	49.73

In vitro analysis of TE protein expression over 72 h

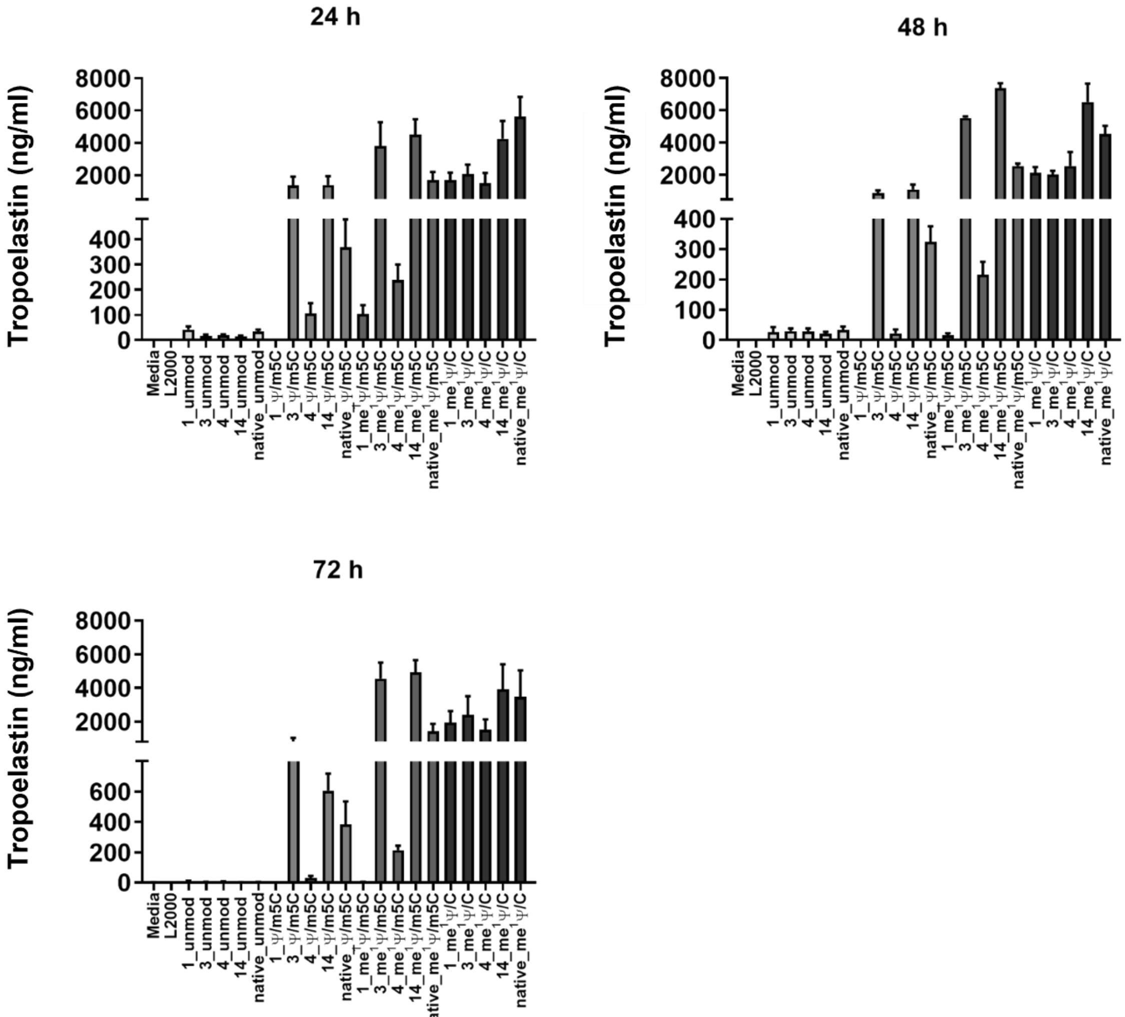

Figure S1: Detection of TE production over time. The influence of different TE mRNA variants on TE synthesis was tested by elastin ELISA. 3x10^5 EA.hy926 cells were transfected with 2.5 µg TE mRNA complexed with 4 µl Lipofectamine 2000 in OptiMEM at 37°C and 5% CO_2 for 4 h. Thereafter, the transfection complexes were replaced with cell culture medium, and the cells were incubated at 37°C and 5% CO_2 without further medium change. The elastin concentration was determined in cell supernatants after 24, 48, and 72 h. As controls, cells were treated with Lipofectamine 2000 (L2000) or OptiMEM only (Medium). The elastin concentration was determined in cell supernatants after 24, 48, and 72 h. Results are shown as mean + SEM (n = 3).

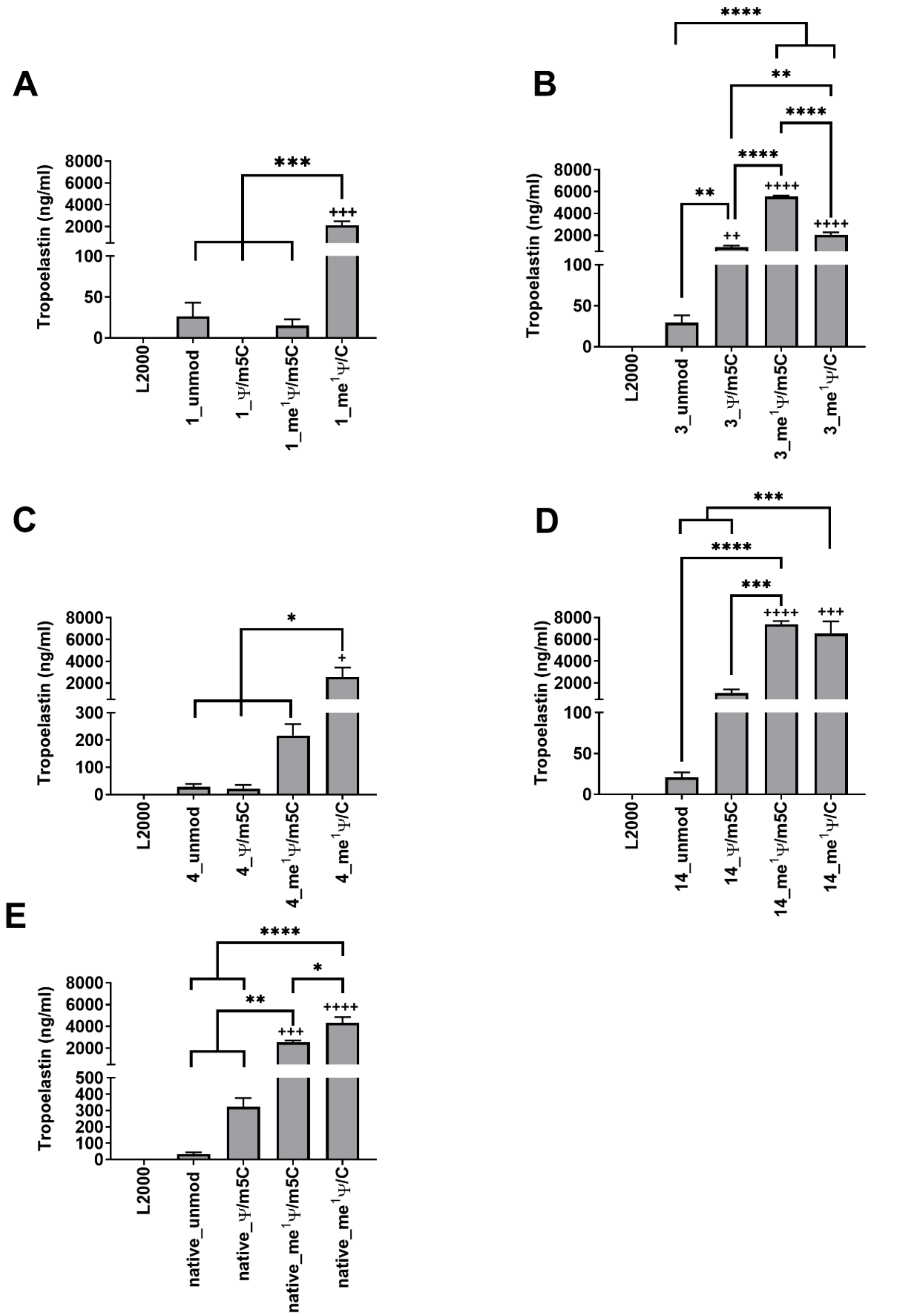

Figure S2: Analysis of TE synthesis after the delivery of TE mRNA into cells. 3×10^5 EA.hy926 cells were transfected with 2.5 µg TE mRNA complexed with 4 µl of Lipofectamine 2000 in OptiMEM for 4 h at 37°C and 5% CO_2. Thereafter, the transfection complexes were replaced by cell culture medium, and the cells were incubated for 48 h at 37°C and 5% CO_2. As a control, cells were treated with

Lipofectamine2000 (L2000) alone. TE concentration was detected in supernatants of cells using elastin ELISA to determine the influence of nucleotide modifications of TE mRNA variants (**A**) 1, (**B**) 3, (**C**) 4, (**D**) 14, and (**E**) native on TE synthesis. The results are shown as mean + SEM (n = 3). Statistical differences were determined using one-way ANOVA followed by Bonferroni's multiple comparisons test. (*p < 0.05, **p < 0.01, *** p <0.001, ****p < 0.0001); + = statistical differences to L2000 control ($^{+}p < 0.05$, $^{++}p < 0.01$, $^{+++}p < 0.001$, $^{++++}p < 0.0001$).

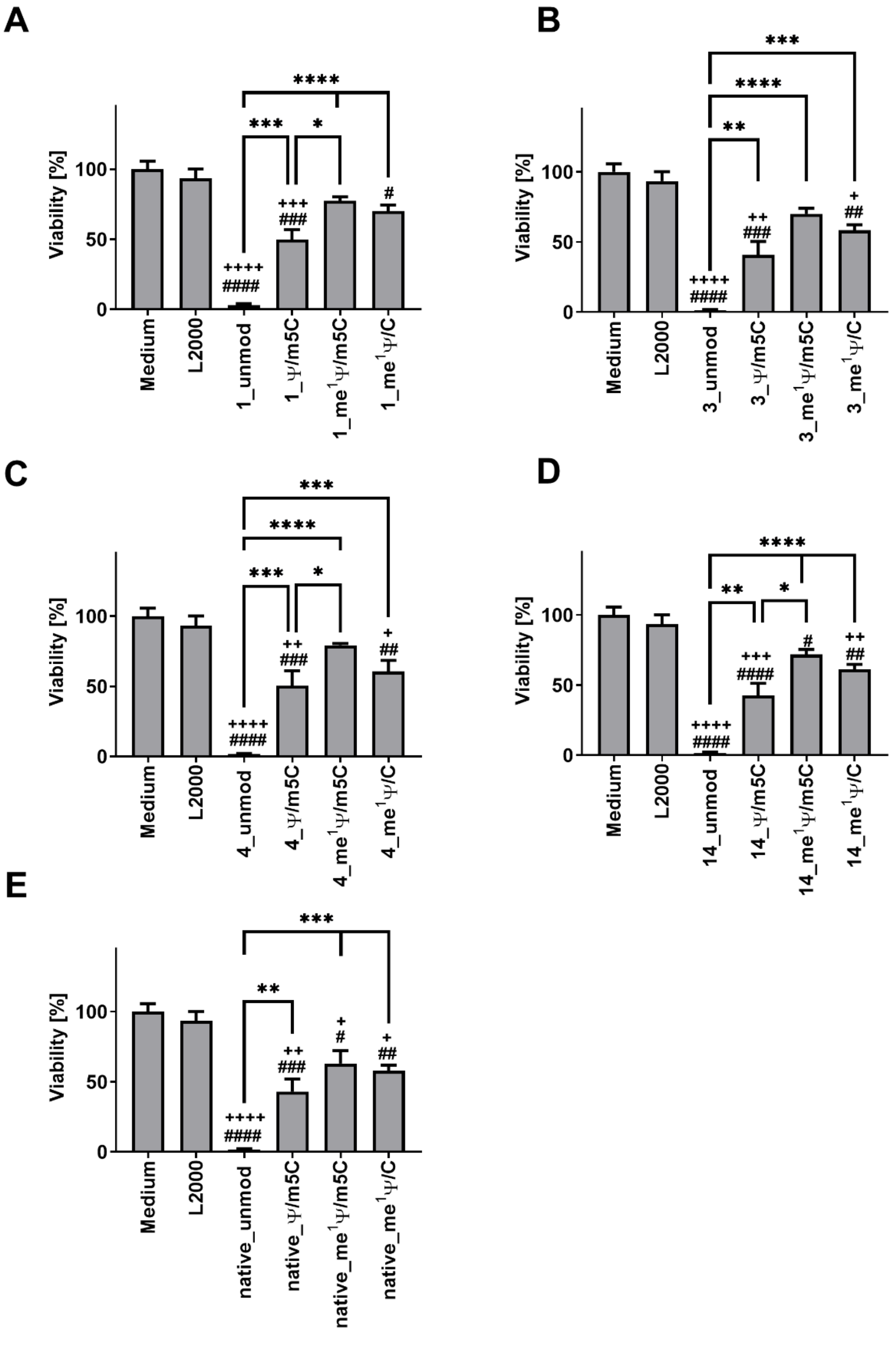

Figure S3: Influence of TE mRNAs on cell viability. $3x10^5$ EA.hy926 cells were transfected with 2.5 µg TE mRNA variants complexed with 4 µl of Lipofectamine 2000 (L2000) in OptiMEM for 4 h at 37°C and 5% CO_2. Thereafter, the transfection complexes were replaced by cell culture medium, and the cells were incubated at 37°C and 5% CO_2. After 24 h, the influence of nucleotide modifications of TE mRNA

variants (**A**) 1, (**B**) 3, (**C**) 4, (**D**) 14, and (**E**) native on cell viability was detected using Presto Blue assay. The viability of cells treated with OptiMEM (medium) was set to 100%. The results are shown as mean + SEM (n = 3). Statistical differences were determined using one-way ANOVA following Bonferroni's comparison test. (* p <0.05, ** p <0.01, *** p <0.001, ****p < 0.0001; # = statistical differences to the medium control ($^{\#}p < 0.05$, $^{\#\#}p < 0.01$, $^{\#\#\#}p < 0.001$, $^{\#\#\#\#}p < 0.0001$); + = statistical differences to L2000 control ($^{+}p < 0.05$, $^{++}p < 0.01$, $^{+++}p < 0.001$, $^{++++}p < 0.0001$).

Analysis of the presence of TE mRNA in the cells

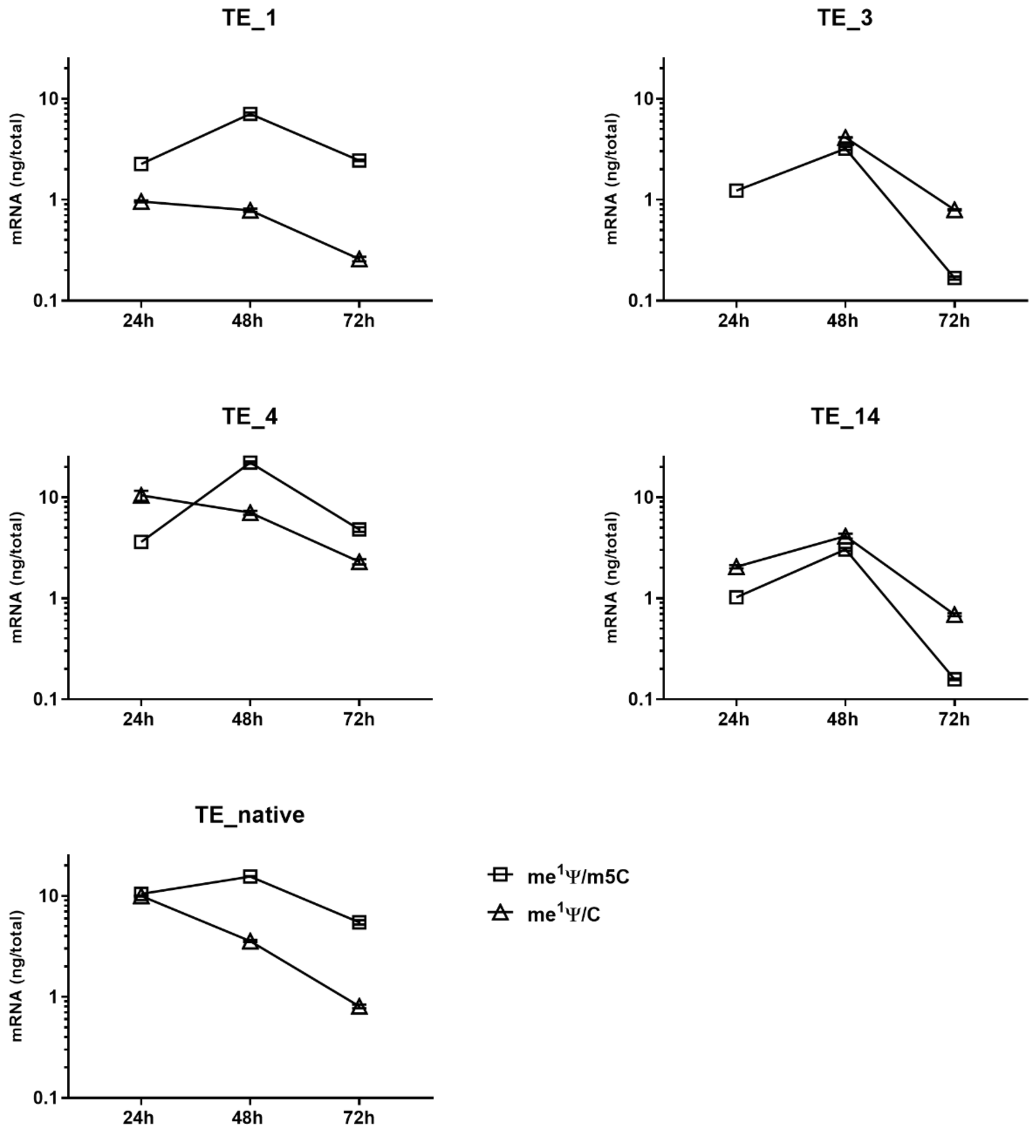

Figure S4: Analysis of the TE mRNA presence in EA.hy926 cells after transfection of TE mRNA variants. The effect of different TE mRNA sequence variants and nucleotide modifications on mRNA

decay was tested by qRT-PCR. Therefore, $3x10^5$ cells were transfected with 2.5 µg TE mRNA complexed with 4 µl of Lipofectamine 2000 in OptiMEM for 4 h at 37°C and 5% CO_2. Thereafter, the transfection complexes were replaced by cell culture medium, the cells were incubated for 2 h at 37°C, 5% CO_2, and the RNA was isolated. Total TE mRNA content was determined by qPCR. Results are shown as mean ± SEM (n = 3).

In vivo application of TE mRNA variants into pig skin by intradermal injection and macroscopic assessment of skin irritation

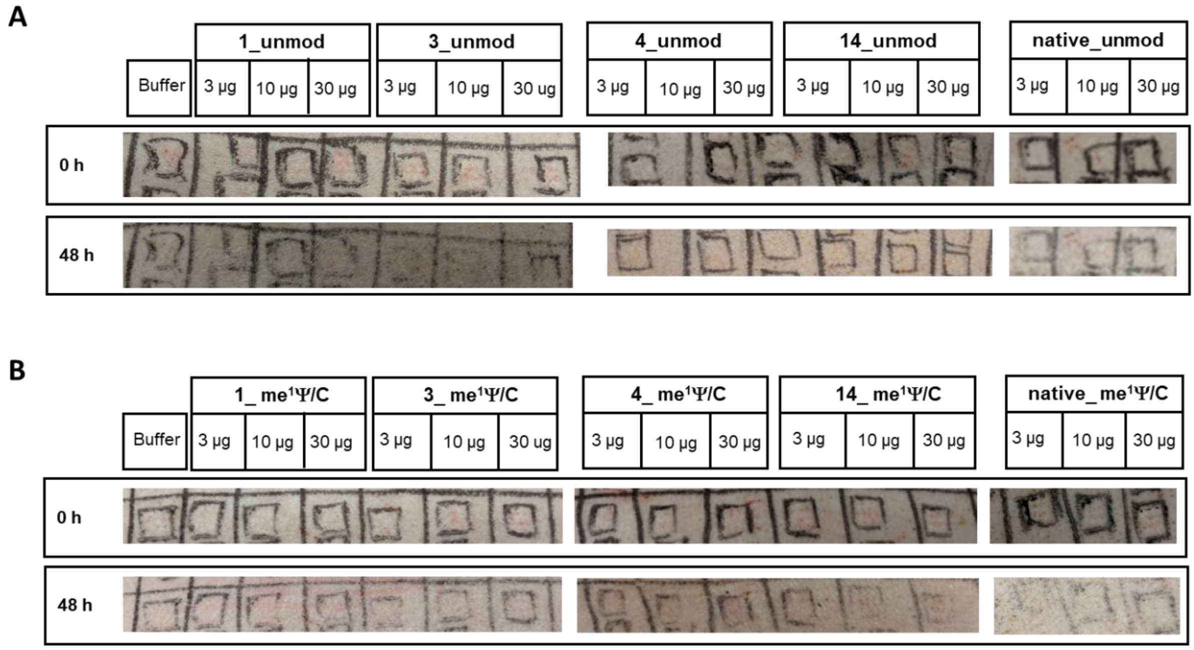

Figure S5: Representative photographic images of porcine skin after in vivo intradermal injection of TE mRNA variants. After injection of 9 x 10 µl Ringer´s lactate buffer without or with 3, 10, or 30 µg TE mRNA variants into a defined skin area of 1x1 cm, the injection sides were marked with a tattoo pen. **(A)** Injection sides of unmodified TE mRNA variants and **(B)** injection sides of $me^1\Psi$/C modified TE mRNA variants after 48 h are shown. No skin irritation or redness was visible.

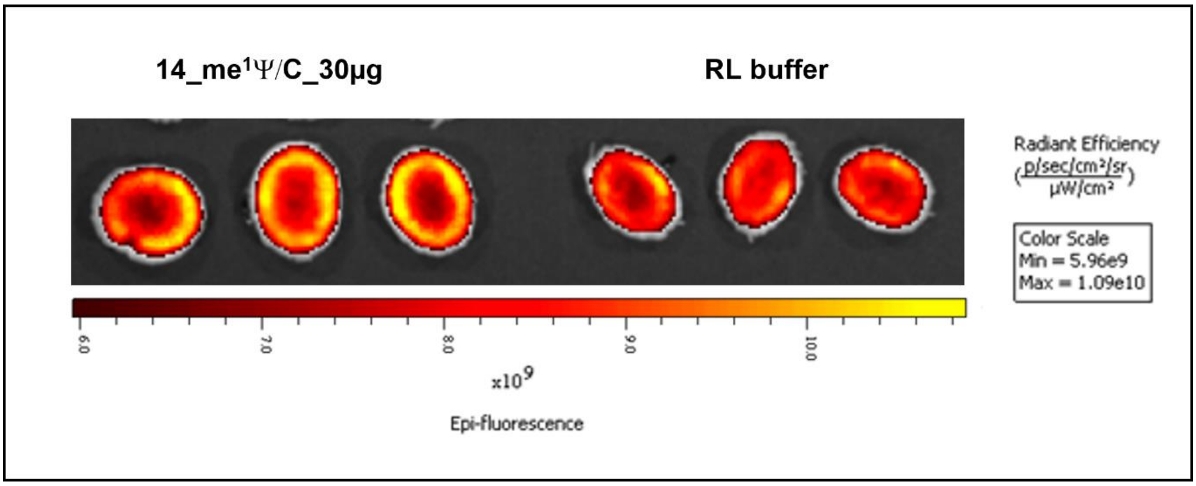

Figure S6: Representative image of IVIS detection of ElaNIR stained porcine skin samples 48 h after the intradermal injection of TE mRNA variants in vivo. 48 h post-injection, skin biopsies injected with 14_me1Ψ/C, corresponding RL buffer only were collected from the injection sites. Biopsies were stained with 20 µM ElaNIR for 30 min. Photographic images including a fluorescent heat map were acquired to indicate fluorescence intensity and distribution areas. Fluorescence emission in a defined region of interest (ROI) was normalized to photons per second per square centimeter per steradian and expressed as average radiant efficiency [p/s/cm2/sr]/[µW/cm^2].

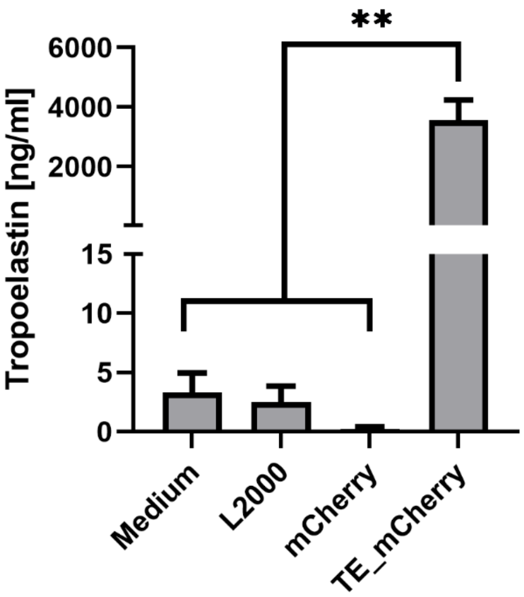

Figure S7: Detection of TE production after TE_mCherry transfection. 3x10^5 EA.hy926 cells were transfected with 2.5 µg mCherry or TE_mCherry mRNA complexed with 4 µl Lipofectamine 2000 (L2000) in OptiMEM at 37°C and 5% CO_2. After 4 h, transfection complexes were replaced with cell

culture medium and the cells were incubated at 37°C and 5% CO_2 for 24 h. Thereafter, the TE concentration was determined in cell culture supernatants using ELISA. As controls, cells were treated with Lipofectamine 2000 (L2000) or OptiMEM only (Medium). Results are shown as mean + SEM (n = 3). Statistical differences were determined using one-way ANOVA following Tukey's comparison test. (** $p < 0.01$).
